# Bavachalcone targets transferrin receptor and sensitizes gemcitabine to affect bladder cancer progression

**DOI:** 10.1002/imt2.70071

**Published:** 2025-08-17

**Authors:** Zihao Zhang, Chenyue Yuan, Qintao Ge, Dalong Cao, Wangrui Liu, Meng Xu, Mengfei Wang, Tao Feng, Yue Wang, Shengfeng Zheng, Zhongyuan Wang, Wei zhang, Xi Tian, Wei Huang, Ziqi Chen, Chao Tu, Hailiang Zhang, Guohai Shi, Jialin Meng, Yijun Shen, Ziliang Wang, Dingwei Ye

**Affiliations:** ^1^ Department of Urology Fudan University Shanghai Cancer Center; Center; Department of Oncology, Shanghai Medical College, Fudan University Shanghai China; ^2^ Qingdao Institute, School of Life Medicine, Department of Urology Fudan University Shanghai Cancer Center, Fudan University Qingdao China; ^3^ Cancer Institute, Shanghai Municipal Hospital of Traditional Chinese Medicine Shanghai University of Traditional Chinese Medicine Shanghai China; ^4^ Department of Urology, Renji Hospital, School of Medicine Shanghai Jiao Tong University Shanghai China; ^5^ Department of Internal Medicine The Third Affiliated Hospital of Soochow University Changzhou Jiangsu China; ^6^ Department of Urology The First Affiliated Hospital of Anhui Medical University Hefei China

**Keywords:** Bavachalcone, bladder cancer, DNA damage repair, gemcitabine resistance, TFRC and RRM1

## Abstract

Gemcitabine resistance drives bladder cancer recurrence and progression. Using high‐throughput drug screening in bladder cancer cells, we identified Bavachalcone (Bava) as a potent gemcitabine sensitizer. Mechanistically, Bava simultaneously targets transferrin receptor (TFRC) and epidermal growth factor receptor (EGFR). It competes with transferrin (Tf) for TFRC binding, reducing cellular iron influx, and inhibits EGFR‐mediated phosphorylation of TFRC at tyrosine 20 (Y20). These actions disrupt mitochondria iron utilization and impairs respiration. The combination of Bava and gemcitabine synergistically inhibits the repair of gemcitabine‐induced DNA damage, while suppressing the iron‐dependent ATR‐CHEK1‐E2F1 pathway and downregulating RRM1 expression. Patient‐derived xenograft models confirmed the superior antitumor efficacy of the Bava‐gemcitabine co‐treatment compared to monotherapies. Clinically, elevated TFRC and RRM1 expression correlates with poor prognosis, supporting their utility as biomarkers of bladder cancer. Our study identified Bava as the first small‐molecule TFRC inhibitor that overcomes gemcitabine resistance through iron modulation, providing both mechanistic insights and a promising therapeutic strategy for bladder cancer.

## Highlights

- Using high‐throughput drug screening and organoid models, we found that Bavachalcone can effectively increase the sensitivity of bladder cancer to gemcitabine.
- Bavachalcone can directly target transferrin receptor (TFRC) and epidermal growth factor receptor, inhibit iron influx and stabilize TFRC on the cell membrane.
- Bavachalcone inhibits the expression of RRM1, a key gemcitabine resistance protein, by suppressing the iron‐dependent ATR‐CHEK1‐E2F1 signaling pathway.
- The PDX model and bladder cancer in situ model were used to verify that Bavachalcone combined with gemcitabine can effectively inhibit the malignant progression of bladder cancer.

## Graphical abstract

Transferrin (Tf) binds extracellular Fe^3+^ and interacts with transferrin receptor (TFRC) on the cell membrane, leading to endocytosis. Iron is reduced by STEAP3, transported by DMT1 into the cytoplasm, then enters mitochondria to support Fe–S cluster formation, mitochondrial respiration, and ATP production. Some Fe^2+^ enters the nucleus, promoting DNA replication and repair via the ATR‐CHEK1‐E2F1 pathway and enhancing RRM1 transcription. Bavachalcone blocks Tf‐TFRC binding and inhibits EGFR‐mediated TFRC phosphorylation, reducing iron uptake. This disrupts mitochondrial function and ATP synthesis, suppresses ATR‐CHEK1‐E2F1 signaling, and downregulates RRM1 transcription.

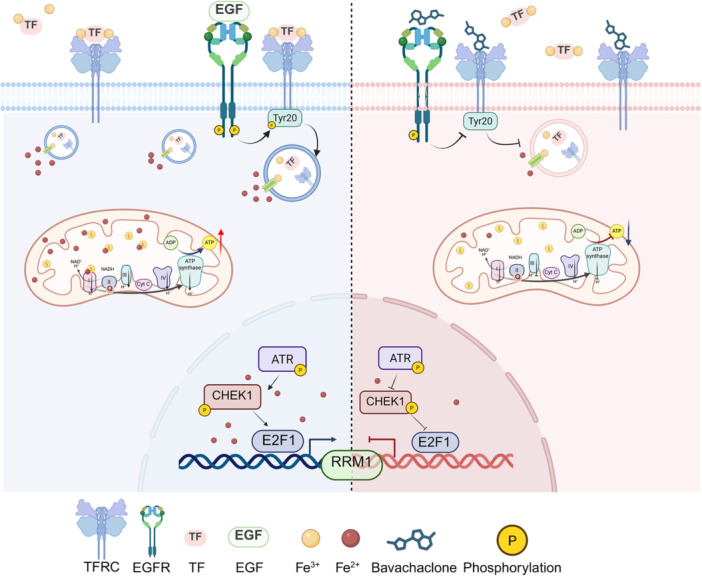

## INTRODUCTION

Bladder cancer (BCa) is the most prevalent urologic malignancy, accounting for over 90% of urothelial tumors and classified into non‐muscle‐invasive (NMIBC, ~75%) and muscle‐invasive (MIBC, ~25%) subtypes [1, 2]. NMIBC is typically managed by transurethral resection of bladder tumors followed by intravesical instillation of Bacillus Calmette‐Guérin or chemotherapeutic agents, such as mitomycin C, doxorubicin, gemcitabine, or thiotepa to reduce recurrence and progression [3]. Despite high initial response rates, up to 60% of NMIBC cases recur, and 10%–20% progress to MIBC. Advanced or refractory tumors require systemic chemotherapy and immunotherapy; neoadjuvant regimens may downstage disease sufficiently to permit radical cystectomy. However, prolonged treatment frequently leads to drug resistance [4].

Gemcitabine, a deoxycytidine analog that inhibits DNA synthesis and induces apoptosis, is a cornerstone of intravesical and systemic chemotherapy for bladder cancer, particularly in BCG‐unresponsive NMIBC [5]. Its efficacy is often limited by resistance, which has been linked to elevated expression of ribonucleotide reductase M1 subunit (RRM1), a key enzyme in deoxyribonucleotide production. Elevated RRM1 levels correlates with gemcitabine resistance across bladder, lung, and pancreatic cancers [6]. RRM1 overexpression reduces gemcitabine incorporation into DNA, increases nucleotide pools, enhances DNA repair capacity, and is associated with poor prognosis and shorter progression‐free survival [7].

Iron homeostasis plays a critical role in cellular function, supporting mitochondrial energy metabolism, oxidative phosphorylation, and nuclear DNA replication and repair. Transferrin receptor (TFRC) mediates ferric ion (Fe^3+^) uptake, which is reduced to ferrous ion (Fe^2+^) by STEAP3 and released into the cytoplasmic via SLC11A2 [8, 9]. Intracellular iron facilitates the formation of iron–sulfur clusters (ISCs), which serve as electron carriers in mitochondrial respiratory complexes and as cofactors for enzymes involved in the TCA cycle, DNA polymerases, helicases, and repair nucleases, thereby maintaining genomic stability [10, 11].

Natural compounds with favorable toxicity profiles are emerging as promising candidates for cancer therapy. Bavachalcone (Bava), a chalcone from *Boehmeria nivea*, exhibits anti‐inflammatory, antioxidant, antibacterial, and anticancer properties by modulating NF‐κB, MAPK, and PI3K/AKT pathways, inducing mitochondrial dysfunction and apoptosis [12, 13]. It also regulates glucose metabolism and has demonstrated tumor growth inhibition effects in preclinical models with favorable bioavailability [14]. However, its molecular targets and mechanisms of action in bladder cancer remain to be elucidated.

Here, through high‐throughput drug screening, we identified Bava as a potent inhibitor of bladder cancer cell proliferation and gemcitabine sensitizer. Mechanistically, Bava concurrently suppresses TFRC and EGFR phosphorylation, disrupting iron uptake and mitochondrial ATP production, while attenuating the ATR‐CHK1‐E2F1 axis and downregulating RRM1 to enhance gemcitabine cytotoxicity. In vitro and in vivo models, including intravesical infusion and patient‐derived xenografts, demonstrate that Bava synergizes with gemcitabine to suppress tumor growth, highlighting its potential as a natural adjunct in bladder cancer therapy.

## RESULTS

### Establishment of cell and patient‐derived organoid (PDO) models for in vitro screening of Chinese medicine monomers

**FIGURE 1:**
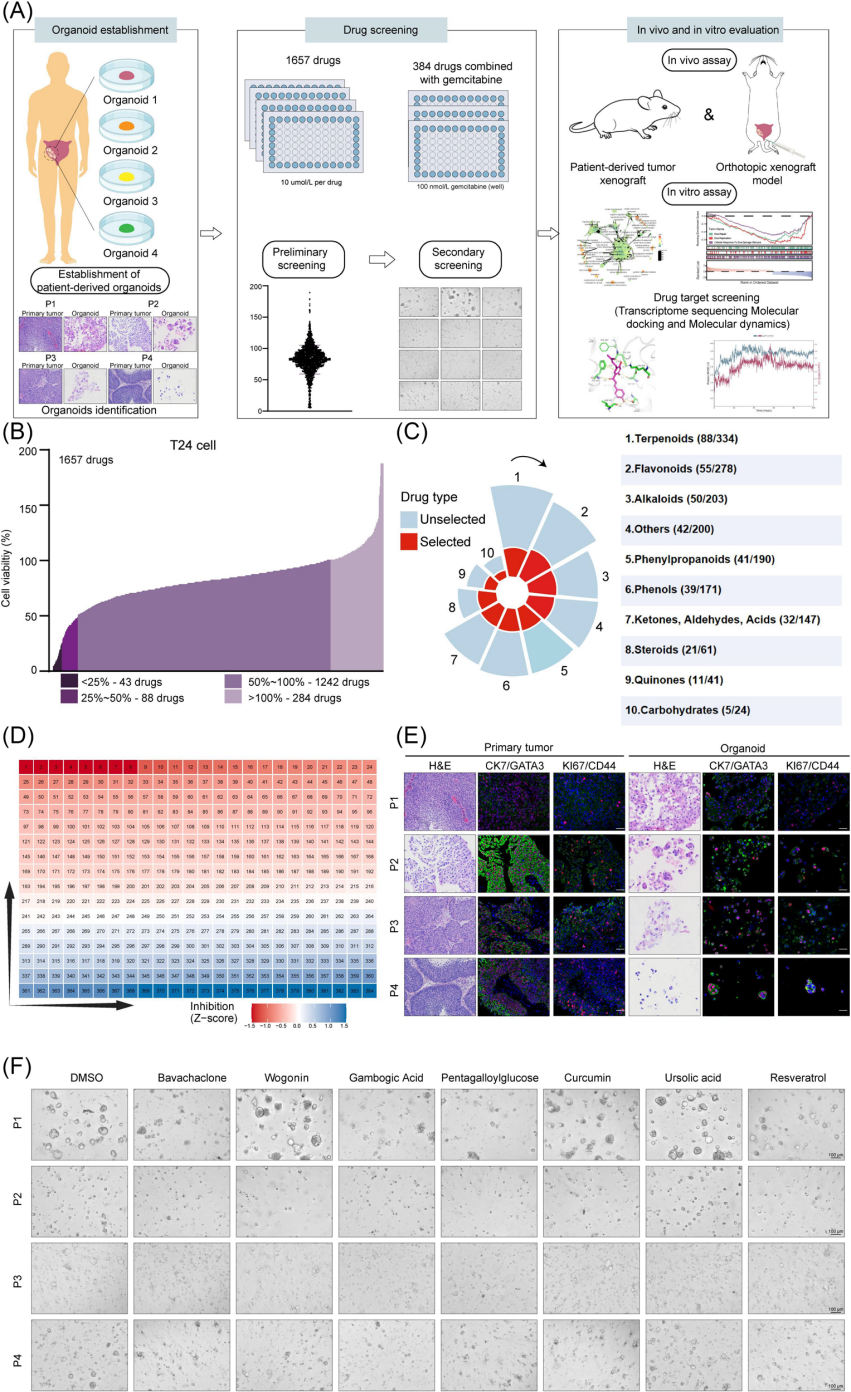
Drug screening based on bladder cancer cells and organoids. (A) Schematic diagram displaying the experimental procedure of drug screening. (B) Preliminary results of high‐throughput drug screening based on bladder cancer cells. (C) Structural classification of drugs in general and selected drugs. The classification of drug candidates was presented in red. (D) Heat map showing the results of the second round of drug screening based on bladder cancer cells. (E) H&E and mIHC staining of four patients' primary tumors (left) and corresponding organoids (right). Scale bars, 50 μm. (F) Representative brightfield images of bladder cancer organoid activity after intervention with different Chinese herbal monomers. Scale bars, 100 μm. mIHC, multiplex immunohistochemistry.

To identify traditional Chinese medicine (TCM) monomers that enhance gemcitabine sensitivity, we implemented a dual screening strategy combining cell‐based and PDO models, validated through subsequent transcriptome sequencing and in vivo studies (Figure 1A). High‐throughput screening of a 1657‐compound TCM monomer library at 10 μM concentration in T24 cells categorized compounds into four groups based on inhibition rates: 43 exhibited >75% inhibition (strong), 88 demonstrated 50%–75% inhibition (moderate), 1242 showed 0%–50% inhibition (weak), and 284 had no significant effect (Figure 1B). Compounds were further classified into 10 structural classes: Terpenoids, Flavonoids, Alkaloids, Phenylpropanoids, Phenols, Ketones‐Aldehydes‐Acids, Steroids, Quinones, Carbohydrates, and Others (Figure 1C). A secondary screen focused on the top 30% inhibitors from each class (384 total), evaluating gemcitabine combination effects. Nine TCM monomers showed significantly enhanced antiproliferative effect on bladder cancer cells when combined with gemcitabine compared to monotherapy (Figure 1D).

Next, we established human bladder cancer organoid cultures following established methods [15]. Fresh tumor tissue from four consented patients was processed into PDO models, with clinical‐pathological characteristics detailed in Table S1. All PDO lines demonstrated stable long‐term expansion (>3 months; passage ratio of 1:2.5–1:3) and faithfully recapitulated parental tumor histopathology (Figure 1E). Multiplex immunohistochemistry confirmed protein expression of biomarkers (GATA3, CK7, KI67, and CD44) in PDOs relative to patient tissues (Figure S1A,B), establishing their utility for drug screening. Through organoid‐based screening, we identified Bava as a potent gemcitabine sensitizer that significantly enhanced drug sensitivity in both cellular and organoid systems (Figure 1F and Figure S1C). While this TCM monomer demonstrates promising chemo‐sensitization activity, its precise inhibitory mechanisms in bladder cancer remain to be elucidated.

### Bavachalcone inhibits malignant biological behaviors of bladder cancer in vitro and in vivo

**FIGURE 2:**
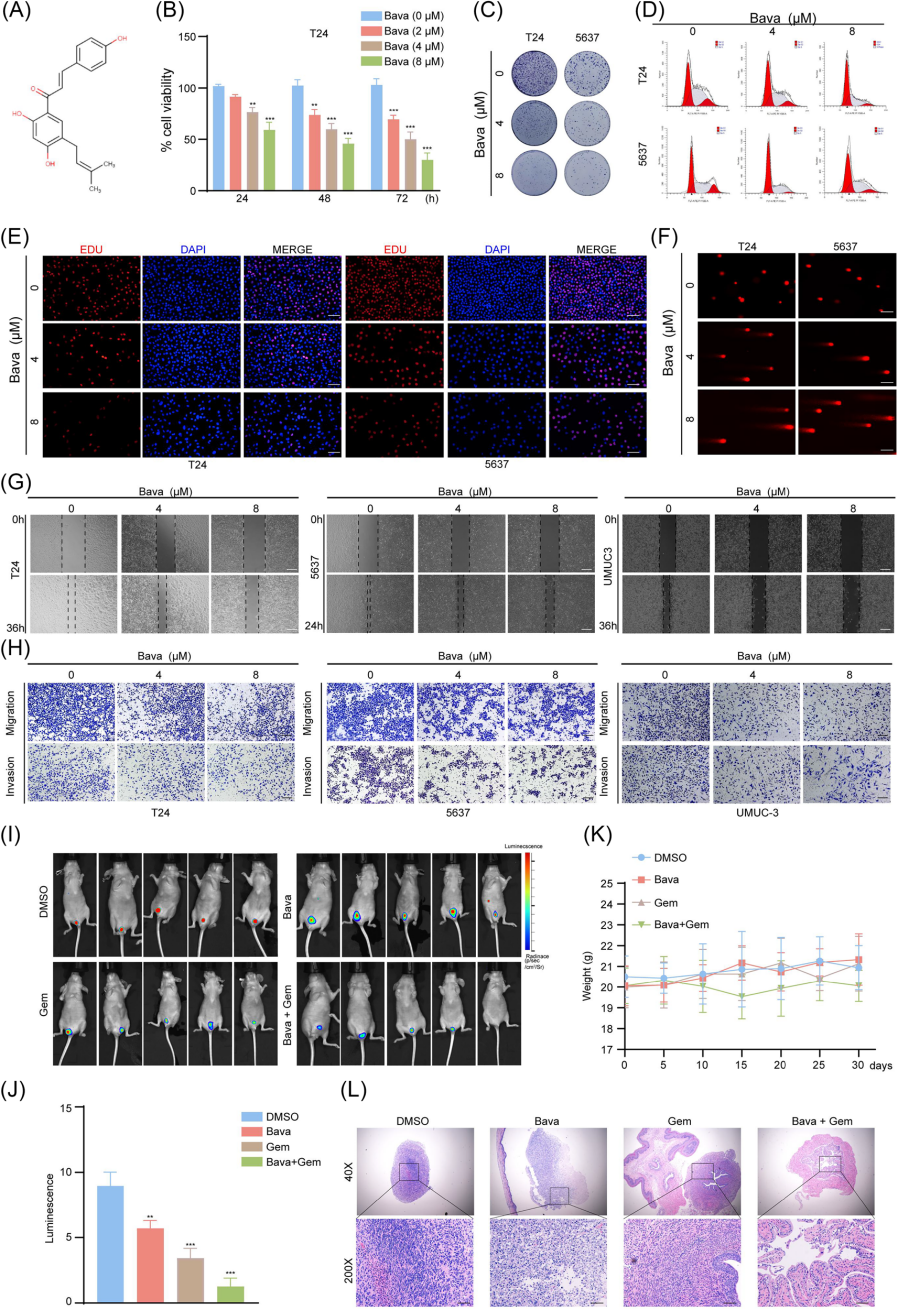
In vitro and vivo studies on the inhibition of bladder cancer by Bavachalcone. (A) Chemical structure of Bavachalcone. (B) The inhibitory effect of Bava on the proliferation of T24 cells after treatment with various concentrations for different time periods. (C) Representative images of colony formation showing T24 and 5637 cells after 24 h of incubation with Bava using a clonogenic assay. (D) Representative flow cytometry histograms showing the cell cycle distribution of T24 and 5637 cells after 24 h treatment with Bava. (E) Representative immunofluorescence images showing DNA replication in T24 and 5637 cells following Bava treatment. Scale bar, 100 μm. (F) Representative immunofluorescence images showing DNA damage in T24 and 5637 cells after 24 h of incubation in Bava. Scale bar, 100 μm. (G) Representative images of the extent of cell healing after 24 h incubation of T24, 5637 and UMUC‐3 cells with Bava using a wound‐healing assay. Scale bar, 100 μm. (H) Representative images of cell invasion and migration of T24, 5637 and UMUC‐3 cells incubated with Bava for 24 h using a transwell assay. Scale bar, 100 μm. (I, J) In vivo imaging showing representative images and quantification of tumor burden in an orthotopic bladder cancer model following treatment with Bava, gemcitabine, or their combination. (K) The weight changes of animals in an orthotopic bladder cancer model over the entire cycle are shown. (L) H&E staining of the orthotopic bladder cancer model. Scale bar, 100 μm. Data represent the mean ± standard deviation (SD) of three replicates. **p < 0.01 and ***p < 0.001.

Figure 2A illustrates the chemical structure of Bava. To evaluate its antitumor efficacy, we conducted a series of in vitro experiments using three bladder cancer cell lines: T24, 5637, and UMUC‐3. Initially, we performed CCK8 assays to determine the IC50 values and assess cell viability at 24‐, 48‐, and 72‐h posttreatment. The results demonstrated that Bava induced a dose‐dependent and time‐dependent reduction in cell viability (Figure 2B and Figure S2A,B). Colony formation assays further confirmed that Bava significantly suppressed the proliferative capacity of T24, 5637, and UMUC‐3 cells (Figure 2C and Figure S2C,D).

Fluorescence‐activated cell sorting analysis revealed that Bava induced cell cycle arrest in the G1‐S phase transition compared to the vehicle control (Figure 2D and Figure S2E,F). This observation was corroborated by cell cycle experiments, which showed that Bava inhibited bladder cancer cells in both G1 and S phases, thereby disrupting DNA synthesis and replication processes. Additionally, EDU incorporation assays, which utilize a thymidine nucleotide analog to evaluate DNA synthesis, demonstrated that Bava decreased EDU binding to DNA, indicating impaired tumor cell proliferation (Figure 2E and Figure S2G,H).

To investigate DNA damage induction, comet assays were performed. Bava‐treated cells exhibited a marked increase in DNA tail moments, indicative of DNA strand breaks (Figure 2F and Figure S2I–J). Furthermore, immunofluorescence analysis of γ‐H2AX, a marker for DNA double‐strand breaks, further confirmed that Bava enhanced γ‐H2AX in bladder cancer cells, reinforcing its role in promoting DNA damage (Figure S2K,L).

Next, the impact of Bava on metastatic potential was assessed via wound healing and Transwell assays. Bava significantly inhibited the migration and invasion of bladder cancer cells (Figure 2G,H and Figure S3A,B).

For in vivo studies, we established a clinically relevant orthotopic bladder cancer model in nude mice using intravesical instillation. While Bava monotherapy demonstrated a limited ability to inhibit tumor progression, combination therapy with gemcitabine resulted in a strong synergistic effect on inhibiting tumor growth (Figure 2I–J). In vivo imaging confirmed that Bava enhanced the therapeutic efficacy of gemcitabine, though Bava alone showed limited antitumor activity compared to gemcitabine. Notably, no significant changes in body weight were observed across treatment groups, suggesting tolerability (Figure 2K). Additionally, histological examination via HE staining revealed that Bava‐gemcitabine combination therapy induced morphological changes in tumor cells, including reduced nuclear atypia and disorder, albeit with persistent vacuolar nuclei, indicating partial alleviation of disease progression (Figure 2L).

Collectively, these in vitro and in vitro findings demonstrate that Bava inhibits the malignant progression of bladder cancer and potentiates the efficacy of gemcitabine, supporting its therapeutic potential.

### Bavachalcone targets TFRC and EGFR

**FIGURE 3:**
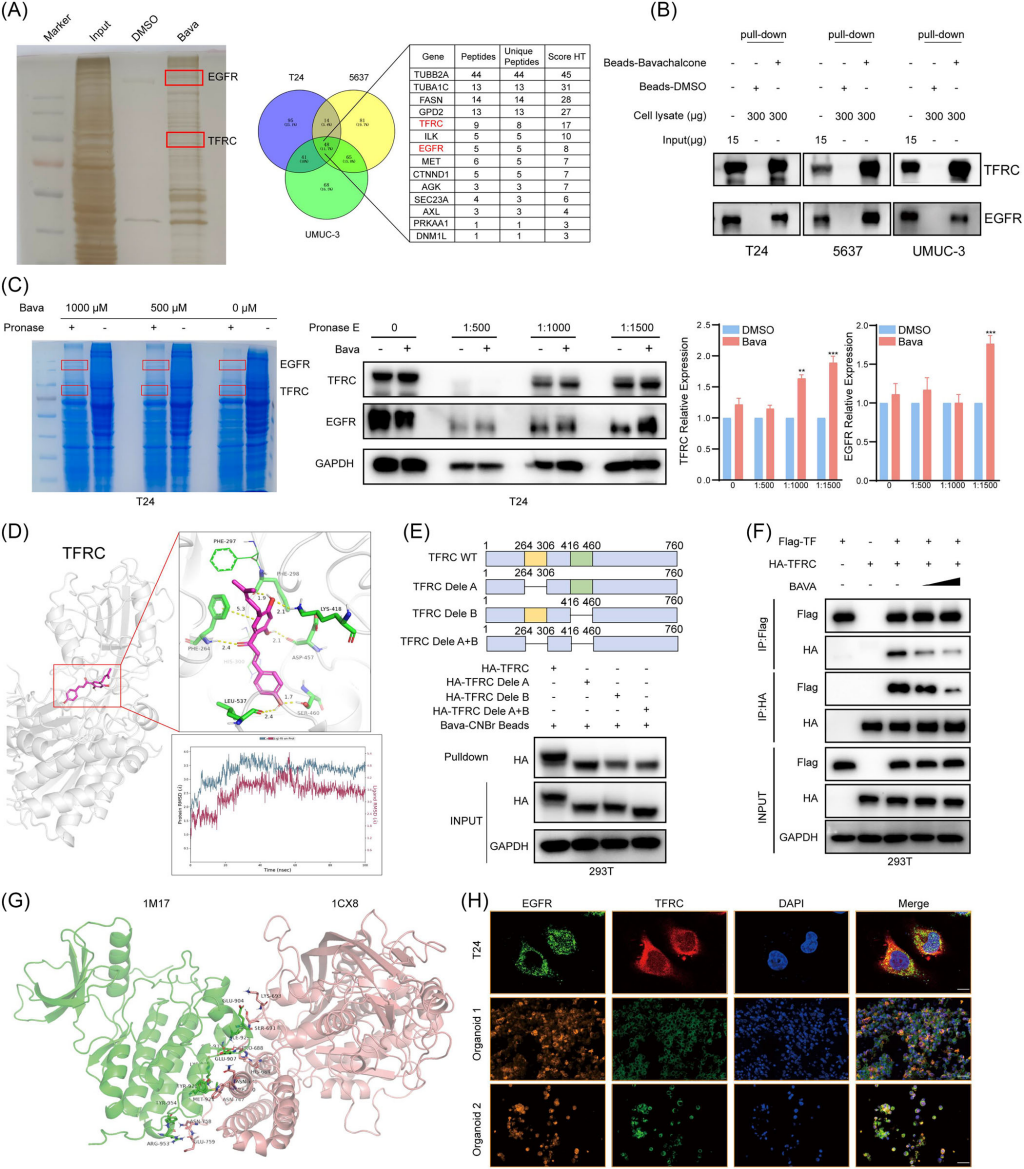
Bavachalcone directly targets TFRC and EGFR and competes with TF for TFRC binding. (A) Silver staining of proteins pulled down with CNBr‐activated Sepharose 4B beads conjugated to Bavachalcone. (B) Representative immunoblot showing that Bavachalcone binds TFRC and EGFR in pulldown assays. (C) Coomassie brilliant blue staining of bladder cancer cell lysates treated with pronase E, with or without Bavachalcone. Representative IB showing that Bava can reduce the ability of pronase E to hydrolyze TFRC and EGFR in the DARTS experiment. (D) Orthogonal view of the Bavachalcone–TFRC complex in the binding pocket, with molecular dynamics simulation results. (E) Pulldown with TFRC deletion mutants (residues 264–306, 416–460, and combined 264–306 + 416–460) to map the Bavachalcone binding sites. (F) Representative immunoblot showing that Bavachalcone inhibits TF–TFRC interaction by competing for TFRC binding. (G) Molecular docking models of TFRC and EGFR interactions (PDB codes 1CX8 and 1M17). (H) Representative immunofluorescence images showing TFRC and EGFR localization in bladder cancer organoids and T24 cells. Scale bars: 25 μm (organoids), 100 μm (T24). Data represent the mean ± SD of three replicates. **p < 0.01 and ***p < 0.001.

To elucidate the mechanism of action of Bava in bladder cancer, we sought to identify its molecular targets. CNBr‐activated Sepharose 4B beads (CS4B), agarose derivatives modified with reactive cyanate esters under alkaline conditions, enable covalent coupling of amine‐containing ligands [16]. Bava was conjugated to CS4B, and pull‐down experiments were performed using lysates from three bladder cancer cell lines. The eluted proteins were resolved on SDS‐PAGE gels, and the analysis revealed specific protein interactions absent in DMSO controls (Figure 3A). Subsequent LC‐MS/MS analysis of excised gel bands identified >100 putative binding partners, with membrane proteins TFRC and EGFR highlighted as key candidates (Table S3). Independent pull‐down assays confirmed direct Bava–TFRC and Bava–EGFR interactions (Figure 3B). Drug Affinity Responsive Target Stability assays demonstrated dose‐dependent protection of TFRC and EGFR from pronase E degradation in Bava‐treated lysates, with Coomassie showing reduced proteolysis (Figure 3C). Immunoblotting confirmed stabilization of both target proteins by Bava (Figure S4A,B). This was further validated by Cellular Thermal Shift Assay (CETSA) [17], in which Bava enhanced the thermal stability of TFRC and EGFR in a temperature‐dependent manner (Figure S4C).

Next, we sought to determine the binding sites of Bava on these two proteins by molecular docking. The analysis predicted Bava binding to the extracellular domains 264–306 and 416–460 of TFRC, and the intracellular domain (742–822) of EGFR. Molecular dynamics simulations confirmed stable binding poses over 100 ns, with Bava maintaining persistent interactions with the TFRC extracellular domains and EGFR intracellular domain (Figure 3D and Figure S4D).

Given the role of TFRC as the primary cellular iron transporter, we investigated whether Bava modulates iron homeostasis. Transferrin (Tf) is an iron‐binding glycoprotein and serves as endogenous ligand for TFRC, with homologous C‐ and N‐terminal domains each binding Fe^3+^ [18]. Tf mediates systemic iron delivery to proliferating cells, transporting iron from intestinal enterocytes, reticuloendothelial macrophages, and hepatocytes [19]. Upon Fe^3+^‐Tf binding, Tf‐TFRC complex undergoes clathrin‐mediated endocytosis into acidified endosomes, triggering iron release.

Given molecular docking predicted potential Bava binding sites at the extracellular domains of TFRC we constructed three TFRC deletion mutants, TFRC‐Δ264–306, TFRC‐Δ416–460 and TFRC‐Δ264–306/Δ416–460, to map the critical binding epitope. Pull‐down assays revealed that deletion of residues 416–460 significantly abolished Bava binding to TFRC (Figure 3E and Figure S4E). CETSA further demonstrated that loss of the 416–460 region, but not the 264–306 domain, impaired Bava‐mediated stabilization of TFRC (Figure S4F), confirming residues 416–460 as the critical binding site.

To determine whether Bava competes with Tf for TFRC binding, we cotransfected 293T cells with Tf and TFRC plasmids and treated them with increasing escalating Bava concentrations. A dose‐dependent reduction in Tf‐TFRC binding was observed (Figure 3F). Fluorescence‐based assays in bladder cancer cells and PDOs confirmed diminished Tf–TFRC interaction post‐Bava treatment (Figure S4G), establishing Bava as a competitive inhibitor of Tf binding.

### Bavachalcone stabilizes membrane‐bound TFRC by inhibiting EGFR‐mediated phosphorylation at tyrosine 20 (Y20)

TFRC internalization represents a crucial step in cellular iron uptake. Previous studies identified Y20 in the TFRC intracellular domain as a key regulatory residue. Notably, TFRC Y20 phosphorylation also confers antiapoptotic properties, enhancing breast cancer cell survival [20]. EGFR, a receptor tyrosine kinase, undergoes ligand‐induced dimerization and autophosphorylation upon EGF binding to its extracellular domain [21]. Emerging evidence suggests that EGFR interacts with TFRC through their intracellular regions [22], promoting us to investigate the potential role of EGFR in TFRC phosphorylation.

Protein docking analysis revealed direct interaction between EGFR and TFRC intracellular domains (Figure 3G), which was validated through co‐localization studies using immunofluorescence microscopy (Figure 3H and Figure S5A). To determine whether EGFR activation regulates TFRC phosphorylation, we treated cells with EGF ± Bava or the EGFR inhibitor gefitinib. EGF stimulation significantly increased both EGFR (Y1078) and TFRC (Y20) phosphorylation, while these effects were abolished by Bava or gefitinib treatment (Figure S5B,C). Subcellular fractionation experiments further demonstrated that the EGF‐induced membrane localization of phosphorylated TFRC and EGFR was similarly suppressed by both Bava and gefitinib (Figure S5D,E). These findings establish a novel mechanism whereby EGFR activation promotes TFRC Y20 phosphorylation.

### Bavachalcone modulates intracellular iron homeostasis and mitochondrial metabolism

**FIGURE 4:**
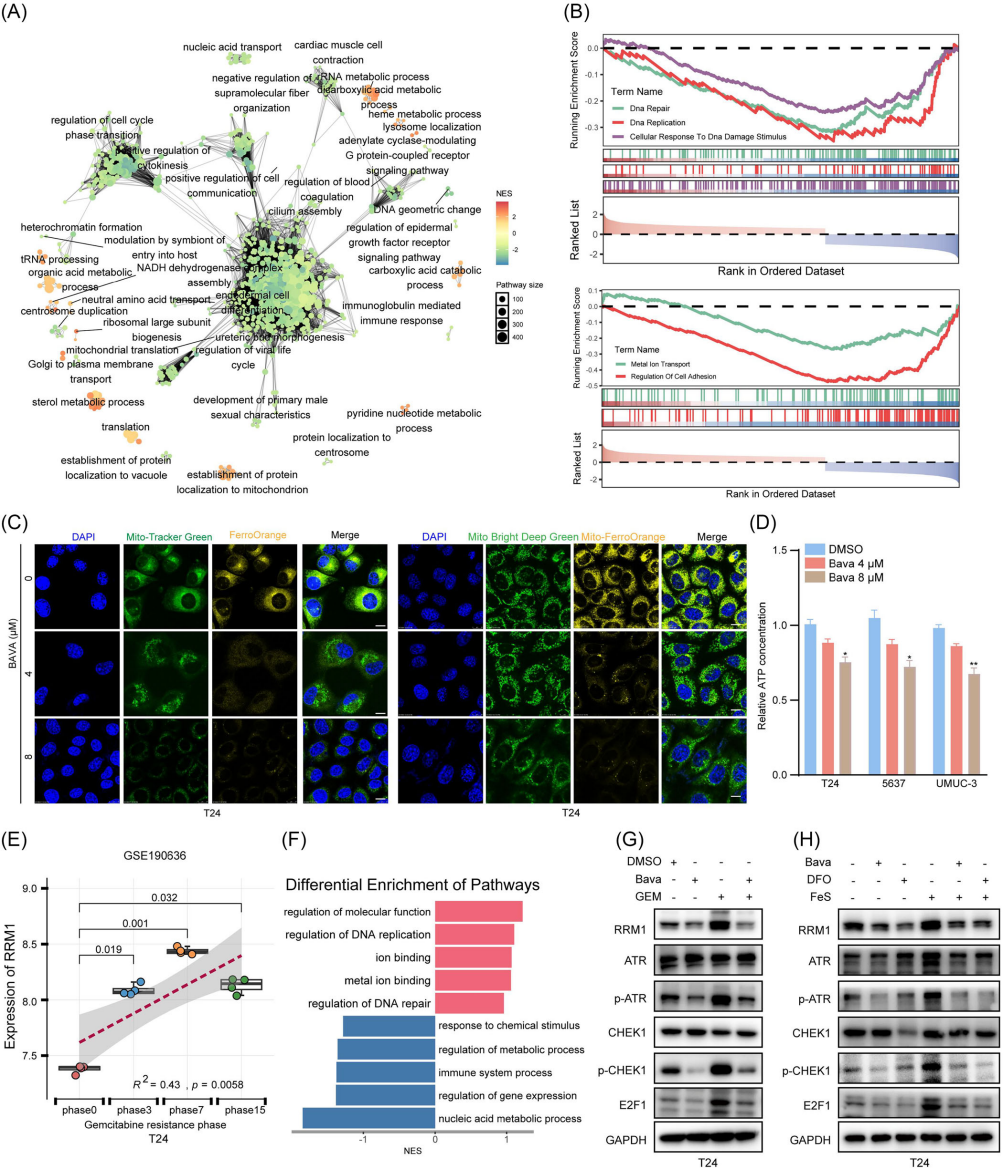
Bavachalcone inhibits DNA damage repair in bladder cancer by suppressing the iron‐dependent ATR‐CHEK1‐E2F1 signaling pathway. (A) GO enrichment analysis of representative genes and pathways altered in T24 cells before and after treatment with Bava. (B) GSEA of representative pathways altered in T24 cells before and after treatment with Bava. (C) Representative immunofluorescence images showing Fe^2+^ levels in the cytoplasm and mitochondria of T24 cells treated with different concentrations of Bava. Scale bar, 25 μm. (D) Intracellular ATP production in T24, 5637, and UMUC‐3 cells treated with various concentrations of Bava. (E) Analysis of RRM1 expression during the progression of gemcitabine resistance using data set GSE190636. (F) GSEA of representative pathways altered in bladder cancer cells with acquired gemcitabine resistance. (G) Representative immunoblot showing ATR‐CHEK1‐E2F1 pathway protein levels after treatment with Bava, gemcitabine, or their combination. (H) Representative immunoblot showing ATR‐CHEK1‐E2F1 pathway protein levels in cells pretreated with ferrous sulfate followed by Bava or deferoxamine (DFO). Data represent the mean ± SD of three replicates. *p < 0.05 and **p < 0.01.

To elucidate the molecular mechanism by which Bava inhibits BCa progression, we performed transcriptome sequencing (RNA‐Seq) to identify differentially expressed genes following Bava treatment. A total of 1248 genes were upregulated and 2264 genes downregulated compared to the DMSO control (Figure S6A; Table S4). Gene Ontology (GO) and Gene Set Enrichment Analyses (GSEA) revealed significant alterations in iron−sulfur cluster binding, metal cluster binding, and GTPase regulator activity post‐treatment. Notably, pathways related to DNA repair, DNA replication, and metal ion transport were suppressed (Figure 4A,B).

Next, we investigated the effect of Bava on iron dynamics by quantifying intracellular total and ferrous iron (Fe^2+^) levels. Bava treatment reduced both total iron and Fe^2+^ concentrations in bladder cancer cells (Figure S6B). Confocal microscopy using a Fe^2+^‐specific probe confirmed decreased Fe^2+^ levels in the cytoplasm and mitochondria (Figure 4C). Fe^2+^, the primary functional form of iron, is critical for biological processes and is transported into mitochondria via SLC25A37 and SLC25A38 to support iron‐sulfur proteins and cytochromes [23]. Given the impact of Bava on mitochondria Fe^2+^, we assessed mitochondrial electron transport chain (ETC) activity. Bava significantly inhibited complexes I, II, and III, but not IV or V (Figure S6C), suggesting disruption of iron‐sulfur protein function. Consequently, ATP synthesis was suppressed in Bava‐treated bladder cancer cells (Figure 4D). These findings indicate that Bava impairs mitochondrial respiration and reduces ATP production by modulating Fe^2+^ levels, thereby contributing to its tumor‐suppressive effects.

To explore the role of TFRC and EGFR in the antitumor mechanism of Bava, we knocked down each protein individually. Bava treatment following TFRC or EGFR knockdown did not further inhibit TFRC phosphorylation (FigureS 7A,B). Furthermore, TFRC or EGFR knockdown combined with Bava treatment did not further inhibit Fe^2+^ uptake, total iron levels, or ATP production (Figure S7C–E). These results imply that Bava inhibits iron influx through TFRC and EGFR, potentially by competing with Tf for TFRC binding and thereby disrupting iron transport.

### Bavachalcone enhances gemcitabine sensitivity by promoting DNA damage repair

GO and GSEA enrichment analyses demonstrated that Bava treatment inhibited bladder cancer cell replication suppressed DNA damage repair signaling (Figure S8A). RRM1 is a crucial enzyme associated with gemcitabine sensitivity, functions as the catalytic subunit of ribonucleotide reductase that generates deoxynucleotides (e.g., dCTP) for DNA synthesis [24]. As a nucleoside analog, gemcitabine inhibits cancer cell proliferation by obstructing DNA synthesis. Elevated RRM1 levels increases intracellular deoxynucleotide pools, diminishing gemcitabine triphosphate availability and reducing therapeutic efficacy [25].

Our quantitative reverse transcription polymerase chain reaction (qRT‐PCR) analysis demonstrated that Bava reduces RRM1 and POLD3 expression in bladder cancer cells (Figure S8B), suggesting its role in impairing DNA replication and repair processes. Further investigation showed that Bava inhibits DNA damage repair by modulating the ATR‐CHEK1 signaling pathway (Figure S8C,D).

To investigate whether Bava could enhance gemcitabine efficacy, we assessed their combined effects on tumor cell growth and DNA damage repair. The Bava‐gemcitabine combination significantly potentiated the antitumor activity of gemcitabine in bladder cancer cells (Figure S9A). Additionally, analysis of the GSE190636 data set showed elevated RRM1 and TFRC expression in gemcitabine‐resistant cells (Figure 4E and Figure S9B). GSEA revealed significant enrichment of metal ion binding and DNA repair pathways during resistance development (Figure 4F).

Mechanistically, we found that gemcitabine treatment activated the ATR‐CHEK1 pathway, upregulating E2F1 and RRM1 expression. This suggests that gemcitabine alone induces DNA replication stress and activates compensatory repair mechanisms that contribute to resistance. Importantly, Bava co‐treatment counteracted these effects, effectively mitigating gemcitabine‐induced DNA damage and suppressing RRM1 expression, thereby restoring chemosensitivity (Figure 4G and Figure S9C).

To elucidate the mechanism of RRM1 upregulation in bladder cancer cells, we interrogated transcription factor databases (ENCODE, CHEA, hTFtarget, TRRUST, GTRD, cor_TCGA, and ChIP_Atlas) and identified E2F1 as a putative transcription regulator of RRM1 (Figure S10A). Genetic silencing of E2F1 significantly reduced both RRM1 mRNA and protein levels in bladder cancer cells (Figure S10B,C), and TCGA data analysis confirmed a strong positive correlation between E2F1 and RRM1 mRNA expression (Figure S10D). Chromatin immunoprecipitation (ChIP) assays verified direct E2F1 binding to the RRM1 promoter (Figure S10E). Through systematic promoter analysis using JASPAR predictions, we divided the RRM1 promoter region into seven domains and identified potential binding sites 800–1100 bp upstream of the transcription start site (Figure S10F,G). Mutagenesis of the two highest‐scoring JARSPAR‐predicted sites (1975–1986 and 904–915 bp) revealed that only the 904–915 bp site was essential for E2F1‐mediated RRM1 transcriptional activation (Figure S10H), suggesting that E2F1 directly enhances RRM1 expression via this region.

Our data have shown that Bava inhibits Tf‐TFRC binding while stabilizing membrane‐bound TFRC, thereby modulating intracellular Fe^2+^ levels. Given the critical roles of Fe^2+^ in mitochondrial and nuclear functions, we investigated whether Fe^2+^ availability influences Bava‐mediated DNA damage repair. Ferrous sulfide (FeS), a widely used iron supplement for treating iron deficiency anemia, was employed as an exogenous Fe^2+^ source, alongside the iron chelator deferoxamine (DFO). FeS treatment activated the ATR‐CHEK1 signaling pathway, upregulating E2F1 and RRM1 expression. Notably, co‐treatment with Bava and DFO effectively alleviated FeS‐induced DNA damage repair and RRM1 expression (Figure 4H and Figure S11A,B), suggesting that Bava modulates RRM1 expression via an iron‐dependent mechanism.

To elucidate the role of the ATR‐CHEK1‐E2F1 axis in Bava‐mediated RRM1 suppression, we individually knocked down ATR, CHEK1, or E2F1 in bladder cancer cells. Each knockdown reduced RRM1 expression and abolished the additional inhibitory effects of Bava. Consistent results were obtained using specific inhibitors (AZD6738 for ATR, Rabusertib for CHEK1, and HLM006474 for E2F1) (Figure S12A–C). These results demonstrate that Bava sensitizes cells to gemcitabine by disrupting ATR‐CHEK1‐E2F1 signaling to suppress RRM1 expression.

### Bavachalcone inhibits bladder cancer PDX growth and sensitizes tumors to gemcitabine

**FIGURE 5:**
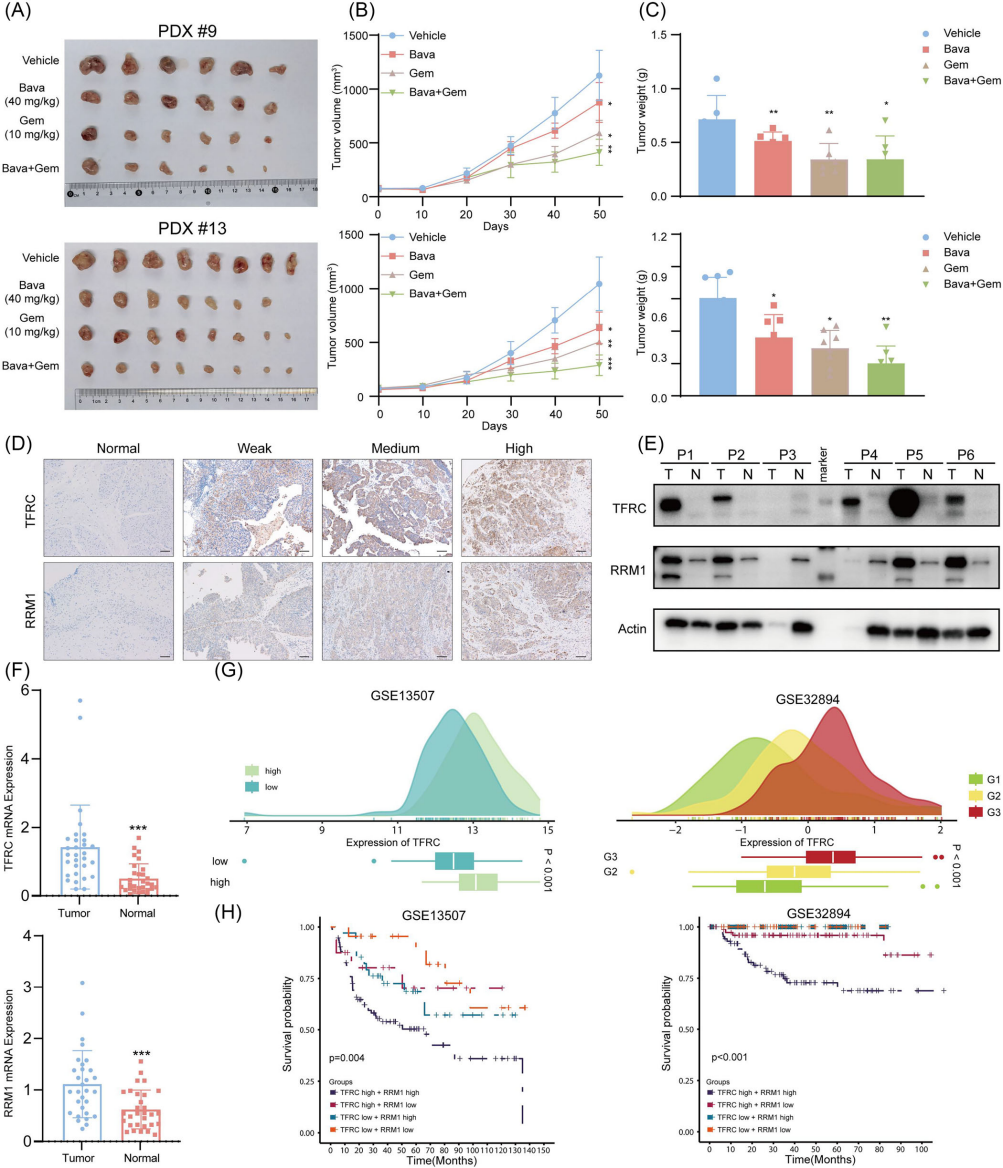
Bavachalcone treatment suppresses bladder tumor growth in PDX models and links TFRC/RRM1 expression to disease progression and patient prognosis. (A) Tumor images excised from the subcutaneous tissue of PDX models after mouse sacrifice. (B) Changes in subcutaneous tumor volume in PDX models over the course of the experiment. (C) Tumor weights of PDX models after excision. (D) Representative IHC images showing the expression of TFRC and RRM1 in normal bladder tissue and different bladder cancer tissues. Scale bar, 100 μm. (E) Representative immunoblot showing TFRC and RRM1 protein levels in paired bladder cancer and adjacent normal tissues. (F) mRNA expression levels of TFRC and RRM1 in unpaired bladder cancer tissues and adjacent normal tissues. (G) TFRC expression levels at different tumor stages in the GSE13507 and GSE32894 datasets. (H) Association of TFRC and RRM1 expression with patient prognosis in the GSE13507 and GSE32894 datasets. Data represent the mean ± SD of three replicates. *p < 0.05, **p < 0.01 and ***p < 0.001.

To evaluate the inhibitory effect of Bava in vivo, we utilized three bladder cancer PDX models (PDX#2, PDX#9, and PDX#13). All PDX tissues were subcutaneously passaged in NSG mice to the third generation before subsequent experiments. Following tumor implantation, mice received intraperitoneal injections of saline (vehicle), Bava, or gemcitabine. Both Bava and gemcitabine significantly inhibited tumor growth compared to the vehicle group. In contrast, the concurrent administration of Bava and gemcitabine yielded further suppression of tumor growth (Figure 5A,B and Figure S13A,B). Notably, the mice received combinatory treatment exhibited no significant changes in body weight (Figure 5C and Figure S13C), hair density, or behavior compared to controls. Tumor assessments confirmed that drug intervention effectively reduced tumor size (Figure S13D). There were no pathological changes observed in the liver and kidneys, as analyzed by hematoxylin and eosin staining (Figure S13E).

### TFRC and RRM1 are poor prognostic markers in BCa

Immunohistochemical (IHC) analysis of 30 bladder cancer specimens from FUSCC demonstrated significantly higher TFRC and RRM1 expression in tumors versus adjacent normal tissues (Figure 5D and FIgure S14A). To validate this, we analyzed 12 paired and 14 unpaired bladder cancer and para‐cancerous tissues via Western blot analysis, confirming elevated TFRC and RRM1 levels in tumors (Figure 5E and Figure S14B). qRT‐PCR results further supported these findings (Figure 5F). Notably, analysis of public datasets (GSE13507 and GSE32894) revealed a positive correlation between TFRC and RRM1 expression and tumor grade progression (Figure 5G and Figure S14C). Prognostic assessment showed that patients with high TFRC or RRM1 levels strongly correlated with worse outcomes, with the poorest survival observed in those co‐expressing both proteins at high levels (Figure 5H and Figure S14D). These results identify TFRC and RRM1 as critical prognostic biomarkers for bladder cancer, with implications for clinical diagnosis and management.

## DISCUSSION

**FIGURE 6:**
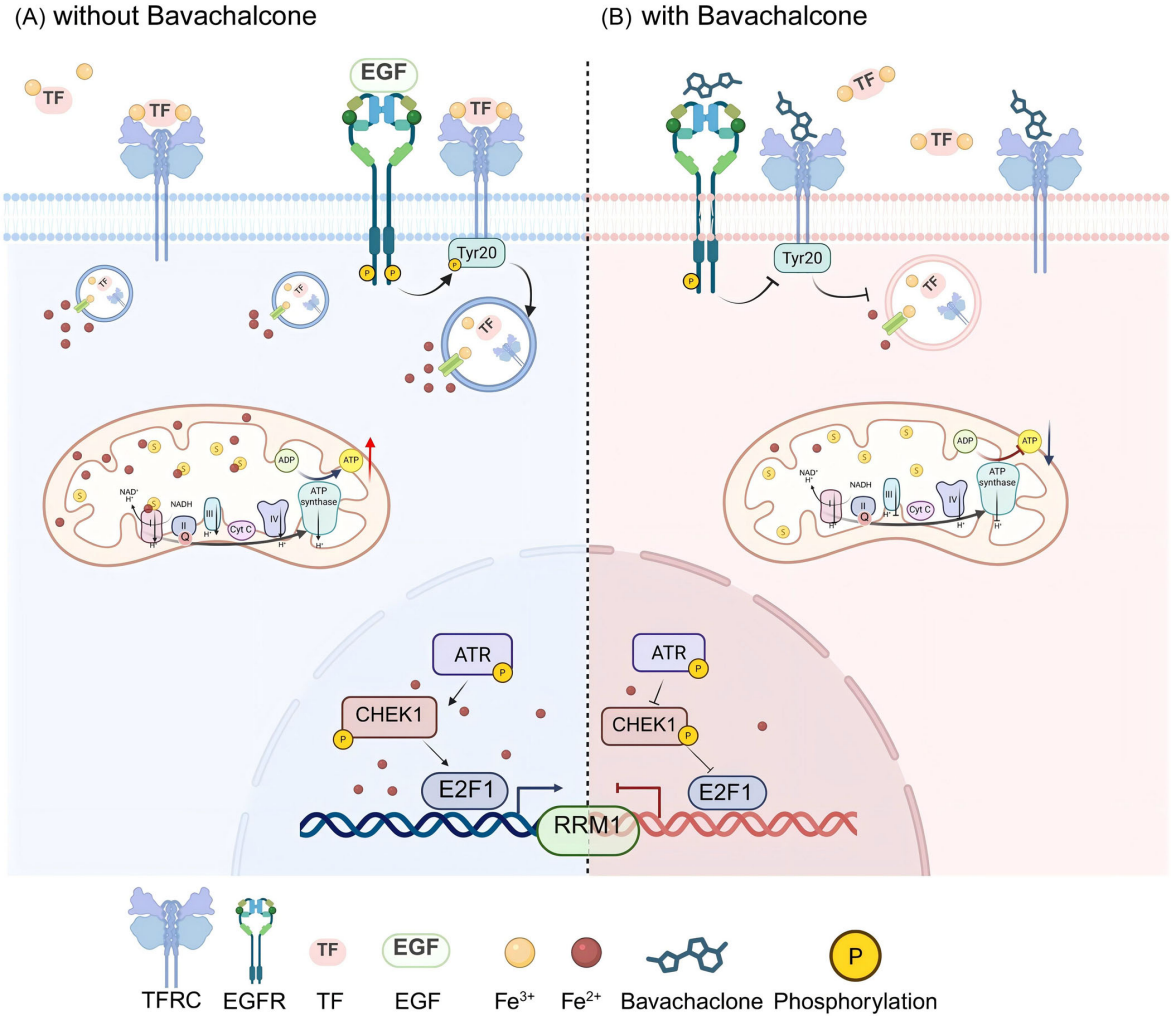
Working model. (A) Tf binds to the extracellular trivalent iron ions, forms a complex, and then binds to TFRC on the cell membrane. It enters the cell through endocytosis, brings the trivalent iron ions into the cell, and is converted into ferrous ions by STEAP3. It is released into the cytoplasm under the action of DMT1, enters the mitochondria through the proteins on the mitochondria and binds to sulfur atoms to form an iron–sulfur complex to help the mitochondrial respiratory chain function and promote ATP generation. Some ferrous ions enter the cell nucleus and participate in the DNA replication and damage repair process. It promotes the expression of the ATR‐CHEK1‐E2F1 signaling pathway and promotes the transcription of RRM1. (B) Bavachalcone blocks the binding of Tf to TFRC and inhibits the phosphorylation of EGFR, thereby inhibiting the phosphorylation of TFRC, causing TFRC to be unable to be internalized and stabilized on the cell membrane, inhibiting the influx of iron ions. It inhibits the activity of the mitochondrial respiratory chain and ATP production. It affects the expression of the ATR‐CHEK1‐E2F1 signaling pathway and inhibits RRM1 transcription.

Gemcitabine, a cornerstone of bladder cancer therapy for nearly three decades, faces growing challenges due to tumor resistance. High‐throughput screening and organoid models identified Bava as a potent gemcitabine sensitizer. In vitro, Bava suppressed bladder cancer cell proliferation and invasion, induced DNA damage, and disrupted iron homeostasis by binding TFRC and EGFR, thereby blocking Tf binding and stabilizing membrane‐bound TFRC. Transcriptomic analyses showed impaired mitochondrial iron import, reduced Fe^2+^ availability, compromised respiratory chain function, and ATP depletion. In the nucleus, Bava inhibited the ATR‐CHK1‐E2F1 axis, downregulating RRM1, a key mediator of gemcitabine resistance. These findings were validated in vivo, with clinical data confirming that elevated TFRC/RRM1 expression correlates with poor prognosis. Collectively, Bava restores gemcitabine efficacy by modulating intracellular iron metabolism (Figure 6).

Iron plays essential roles throughout evolution, serving as a crucial cofactor in fundamental biological processes including respiration, DNA synthesis, and repair [26, 27]. In mammalian systems, dietary ferric ions bind Tf and enter cells via TFRC‐mediated endocytosis. Subsequent reduction by STEAP3 and transport through SLC11A2 releases Fe²⁺ for cellular utilization [28]. Our findings show that Bava binds the 406–460 region of TFRC, competitively inhibiting Tf‐TFRC interaction. Simultaneously, it prevents EGFR‐mediated phosphorylation of TFRC at Tyr20.

Mitochondria rely on Fe²⁺ imported via SLC25A37 and SLC25A38 to synthesize ISCs [23, 29]. ISCs function as a multifunctional cofactor essential for numerous biochemical reactions, ensuring the smooth progression of the tricarboxylic acid cycle and the ETC [30]. Mitochondrial respiratory chain complexes require at least one ISC, heme, or a combination of both to facilitate electron transfer activity and regulate ATP production [31]. ETC damage activates reactive oxygen species, promoting tumor progression. In this study, Bava reduced mitochondria Fe²⁺ content and decreased the activity of complexes I, II, and III, thereby inhibiting ATP production. These findings indicate that Bava exerts tumor‐suppressive effects by disrupting electron transport and ATP production.

The nucleus is the site of DNA replication, where the ribonucleotide reductase (RNR) enzyme complex, composed of catalytic RRM1 and iron‐dependent RRM2/RRM2B subunits, converts ribonucleotides into deoxyribonucleotides, a rate‐limiting step in DNA synthesis [24, 32, 33]. Iron chelation inhibits RNR, while CCND1‐CDK4/6‐mediated RB phosphorylation releases E2F1 to drive RRM1 transcription [34, 35]. We show that Bava suppresses CDK4/6 expression and blocks ATR‐CHK1‐E2F1 signaling, resulting in reduced RRM1 expression. In contrast, exogenous FeS or gemcitabine treatment activates this pathway to promote DNA repair. Importantly, Bava reverses FeS‐ and gemcitabine‐induced pathway activation and further downregulates RRM1, impairing tumor cell DNA damage responses.

Despite advances, the role of iron metabolism in chemoresistance remains incompletely understood due to the complexity and multitude of regulatory proteins of the pathway. Combining natural products with chemotherapy offers a promising strategy to overcome resistance, reduce drug doses, and minimize toxicity. Building on extensive reports of phytochemicals enhancing chemotherapeutic efficacy, we will continue dissecting the iron‐chemoresistance axis in bladder to guide future drug development.

## CONCLUSION

This study elucidates the multifaceted mechanism by which Bava enhances gemcitabine sensitivity in bladder cancer. We demonstrate that Bava directly binds TFRC, reducing cellular iron uptake. This disruption cascades to mitochondrial dysfunction, including impaired ISC synthesis, comprised ETC activity, and ATP depletion. Concurrently, Bava suppresses the ATR‐CHK1‐E2F1‐RRM1 signaling axis, downregulating RRM1 expression. By targeting both mitochondria iron metabolism and nuclear DNA repair pathways, Bava restores the antitumor efficacy of gemcitabine. These findings advance our understanding of tumor metabolism and offer a promising strategy to sensitize resistant cancers to chemotherapy, with implications extending beyond bladder cancer.

## METHODS

### Patients sample

Thirty BCa clinical samples were obtained from the Fudan Shanghai Cancer Center. The use of human bladder cancer samples and the clinical parameters were approved by the Institutional Review Board of Fudan Shanghai Cancer Center (050432‐4‐2108).

### Cells and reagents

HEK‐293T (ZQ0033), T‐24 (ZQ0120), 5637 (ZQ0114), and UM‐UC‐3 (ZQ0347) cell lines were obtained from Shanghai ZhongQiao Xin Zhou Biotechnology Co., Ltd. All cells were incubated according to the recommended protocol. Bavachalcone (B20117) (purity ≥ 98%) was provided by Shanghai Yuanye Bio‐Technology Co., Ltd. Deferoxamine Mesylate (DFO) (purity ≥ 99.80%), Gefitinib (purity ≥ 99.92%), AZD6738 (purity ≥ 99.99%), Rabusertib (purity ≥ 99.63%), HLM006474 (purity ≥ 99.04%), and Gemcitabine (purity ≥ 99.67%) were purchased from TargetMol. The primary antibodies and other reagents used in this study are detailed in Table S2.

### Establishment of patient‐derived tumor organoids

BCa specimens were provided by the Fudan Shanghai Cancer Center. The bladder cancer samples used to construct the organoid model were all specimens from patients undergoing transurethral resection. They had not undergone any chemotherapy or immunotherapy before surgery. Their pathological types were all transitional epithelial cell carcinoma. Fresh tumor samples were stored in the antibiotic‐containing DMEM medium with 10 μM Y‐27632 and 10% fetal bovine serum after surgically resected and transported to the laboratory at 4°C for immediate processing. Briefly, after being washed gently at least three times with pre‐chilled 1 × DPBS with 3% Penicillin‐Streptomycin Solution, tumor tissues were cut into small pieces using surgical scissors and digested with tissue digestion (D23013, D1Med) to obtain single‐cell suspensions. After digestion, the suspension was passed through a 100 μm cell strainer to remove undigested parts, and then centrifuged at 500 × *g* for 5 min. The pellet was resuspended with Matrigel (D23016, D1Med) and dispensed into a 24‐well cell culture dish. After 15 min of solidification of Matrigel, conditioned medium was then added. Conditioned medium was provided by D1 Medical Technology Company (K21111).

## AUTHOR CONTRIBUTIONS

**Zihao Zhang**: Writing—original draft; methodology; data curation; conceptualization. **Chenyue Yuan**: Data curation; Writing—original draft; validation. **Qintao Ge**: Software; validation; investigation. **Dalong Cao**: Conceptualization; project administration. **Wangrui Liu**: Resources; supervision. **Meng Xu**: Validation; investigation. **Mengfei Wang**: Formal analysis; data curation. **Tao Feng**: Methodology. **Yue Wang**: Methodology; visualization. **Shengfeng Zheng**: Visualization. **Zhongyuan Wang**: Resources. **Wei Zhang**: Methodology. **Xi Tian**: Methodology. **Wei Huang**: Investigation. **Ziqi Chen**: Investigation; validation. **Chao Tu**: Formal analysis. **Hailiang Zhang**: Supervision. **Guohai Shi**: Supervision; visualization. **Jialin Meng**: Project administration; conceptualization; resources. **Yijun Shen**: Project administration; resources; conceptualization; methodology; funding acquisition. **Ziliang Wang**: Methodology; conceptualization; funding acquisition; project administration; supervision. **Dingwei Ye**: Supervision; conceptualization; project administration; funding acquisition.

## CONFLICT OF INTEREST STATEMENT

The authors declare no conflicts of interest.

## ETHICS STATEMENT

This study received approval from the ethics committee of the Institutional Review Board of Fudan Shanghai Cancer Center (050432‐4‐2108) and the Laboratory Animal Welfare and Ethics Committee of Fudan Shanghai Cancer Center (Approval No. FUSCC‐IACUC‐S2024‐0541).

## Supporting information


**Figure S1.** Bladder cancer organoid mIHC staining.
**Figure S2.** In vitro study on the inhibition of bladder cancer by Bavachalcone.
**Figure S3.** In vitro study on the inhibition of bladder cancer by Bavachalcone.
**Figure S4.** Bavachalcone directly targets TFRC and EGFR.
**Figure S5.** Bavachalcone inhibits EGF‐induced EGFR and TFRC phosphorylation.
**Figure S6.** Bavachalcone inhibits iron influx and mitochondrial respiratory chain activity in bladder cancer.
**Figure S7.** Bava could not further inhibit the phosphorylation of EGFR and TFRC after knockdown of EGFR and TFRC.
**Figure S8.** Bavachalcone inhibits DNA damage repair in bladder cancer.
**Figure S9.** Bavachalcone combined with gemcitabine inhibits DNA damage repair in bladder cancer.
**Figure S10.** Bavachalcone combined with DFO or FeS affects the iron‐dependent ATR‐CHEK1‐E2F1 signaling pathway.
**Figure S11.** E2F1 regulates the transcription of RRM1.
**Figure S12**. Knockdown or pharmacological inhibition of ATR–CHEK1–E2F1 signaling modulates Bavachalcone's pathway suppression.
**Figure S13.** Bavachalcone inhibits progression in bladder cancer PDX models.
**Figure S14.** TFRC and RRM1 are poor prognostic indicators in bladder cancer.

**Table S1.** Patient Information.
**Table S2.** Antibody, reagent information, and primer information.
**Table S3.** Mass spectrometry.
**Table S4.** RNA‐seq.

## Data Availability

The data that support the findings of this study are openly available in imeta.project at https://github.com/zhangzh-11/imeta.project. The correlation, expression, and prognostic data were obtained from the GEO database (GSE190636, GSE13507, and GSE32894). The data and scripts used are saved in GitHub (https://github.com/zhangzh-11/imeta.project). Supplementary materials (methods, figures, tables, graphical abstract, slides, videos, Chinese translated version, and update materials) may be found in the online DOI or iMeta Science http://www.imeta.science/.
