# Supplementary material for: Bavachalcone targets transferrin receptor and sensitizes gemcitabine to affect bladder cancer progression
Source: Imeta. 2025 Aug 17;4(5):e70071. doi: 10.1002/imt2.70071 (PMC12527993; doi:10.1002/imt2.70071)
Supplement: Supplementary file 1 — Figure S1. Bladder cancer organoid mIHC staining. Figure S2. In vitro study on the inhibition of bladder cancer by Bavachalcone. Figure S3. In vitro study on the inhibition of bladder cancer by Bavachalcone. Figure S4. Bavachalcone directly targets TFRC and EGFR. Figure S5. Bavachalcone inhibits EGF‐induced EGFR and TFRC phosphorylation. Figure S6. Bavachalcone inhibits iron influx and mitochondrial respiratory chain activity in bladder cancer. Figure S7. Bava could not further inhibit the phosphorylation of EGFR and TFRC after knockdown of EGFR and TFRC. Figure S8. Bavachalcone inhibits DNA damage repair in bladder cancer. Figure S9. Bavachalcone combined with gemcitabine inhibits DNA damage repair in bladder cancer. Figure S10. Bavachalcone combined with DFO or FeS affects the iron‐dependent ATR‐CHEK1‐E2F1 signaling pathway. Figure S11. E2F1 regulates the transcription of RRM1. Figure S12. Knockdown or pharmacological inhibition of ATR–CHEK1–E2F1 signaling modulates Bavachalcone's pathway suppression. Figure S13. Bavachalcone inhibits progression in bladder cancer PDX models. Figure S14. TFRC and RRM1 are poor prognostic indicators in bladder cancer. [file IMT2-4-e70071-s002.docx]

**Supporting information to**

**Bavachalcone targets transferrin receptor and sensitizes gemcitabine to affect bladder cancer progression**

**Running title：Bavachalcone Sensitizes Bladder Cancer to Gemcitabine via Transferrin Receptor Targeting**

Zihao Zhang^1,2#^, Chenyue Yuan^3#^, Qintao Ge^1,2#^, Dalong Cao^1#^, Wangrui Liu^4^ Meng Xu^3^, Mengfei Wang^3^, Tao Feng^1,2^, Yue Wang^1^, Shengfeng Zheng^1^, Zhongyuan Wang^1^, Wei zhang^1^, Xi Tian^1,^, Wei Huang^3^, Ziqi Chen^3^, Chao Tu^5^, Hailiang Zhang^1^, Guohai Shi^1^, Jialin Meng^6*^, Yijun Shen^1*^, Ziliang Wang^3*^ , and Dingwei Ye^1*^

1. Department of Urology, Fudan University Shanghai Cancer Center, Fudan University, Shanghai, 200032, China

2. Qingdao Institute, School of Life Medicine, Department of Urology, Fudan University Shanghai Cancer Center, Fudan University, Qingdao 266500, China

3. Cancer Institute, Shanghai Municipal Hospital of Traditional Chinese Medicine, Shanghai University of Traditional Chinese Medicine,Shanghai 200071, China

4. Department of Urology, Renji Hospital, School of Medicine, Shanghai Jiao Tong University, Shanghai, 200025, China.

5. Department of Internal Medicine, The Third Affiliated Hospital of Soochow University, Changzhou, Jiangsu, 213000, China

6. Department of Urology, The First Affiliated Hospital of Anhui Medical University, Hefei, 230032, China

# These authors contributed equally: Zihao Zhang, Chenyue Yuan, Qintao Ge, Dalong Cao

* Correspondence: dwyelie@163.com (Dingwei Ye), wangziliang@shutcm.edu.cn (Ziliang Wang ), yijunshen79@163.com (Yijun Shen) & mengjialin@ahmu.edu.cn(Jialin Meng)

Supplementary Materials:

1. Detailed Methods

2. Supplementary Figures

3. Table S1 Patient Information

4. Table S2 Antibody, reagent information and primer information

5. Table S3 RNA-seq

6. Table S4 Mass spectrometry

**Detailed Methods**

**DNA construction and mutagenesis**

Polymerase chain reaction (PCR)-amplified human TFRC, Tf and EGFR were cloned into pcDNA3.1-HA or pcDNA3.1-Flag vector. TFRC mutant (Delete 264-306 and 416-460) were generated using the Mut Express II Fast Mutagenesis Kit V2 (Vazyme, China).

**Cell toxicity and proliferation assay**

Cell viability and proliferation assays were performed as described previously[16, 17]. The cell number was assessed by CCK8 after treatment with Bavachaclone and Gemcitabine for 24, 48 or 72 h.

**High-throughput drug screening**

The Chinese medicine monomer compound library was purchased from MCE.Spread an appropriate amount of T24 cells in a 96-well plate, add 10 μM traditional Chinese medicine monomer to each well after attachment, and detect the activity of T24 cells by CCK8 after 24 h. Mix Gemcitabine and culture medium in advance so that the concentration of Gemcitabine in culture is 100 nM. After the cells adhere to the wall, discard the original culture medium and add new culture medium containing Gemcitabine. Then add 10 μM Chinese medicine monomer to each well. After 24 hours, pass through CCK8 detect T24 cell activity.

**Colony formation assay**

Cells were seeded in 6-well plates for 24 h. Cells were then treated with Bava at different concentrations and incubated in a humidified incubator at 37 °C for 48 h. Continue incubation in the incubator for 10 days. Wash with PBS 3 times, fix with 4% paraformaldehyde for 1 h, then wash with PBS 3 times. Stained with crystal violet for 1 h, washed three times with PBS.

**EDU assay**

Cells were seeded in 6-well plates for 24 h. Cells were then treated with Bava at different concentrations and incubated in a humidified incubator at 37 °C for 24 h. Prepare 10 μM EDU working solution and stain at 37 °C for 2h. Wash with PBS, fix with 4% paraformaldehyde, permeate with PBS containing 0.3% Triton X-100, and then block. Prepare the EDU staining solution according to the instructions and stain for 30 min. Wash with PBS and stain with DAPI for 15 min. After washing three times, use a fluorescence microscope to observe and take pictures (C0085, Beyotime).

**Animals**

All experimental procedures using mice were performed in accordance with protocols approved by Laboratory Animal Welfare and Ethics Committee of Fudan shanghai cancer center (Approval No. FUSCC-IACUC-S2024-0541). Athymic BALB/c nude mice (Strain NO.D000521)(5-week-old) were purchased from GemPharmatech company and bred under specific pathogen-free conditions. Use isoflurane to anesthetize nude mice, put a 24G indwelling needle soaked in paraffin oil into the bladder through the urethra, inject 100 μL of 0.15 M AgNO_3_, let it stand for 10 s, use 100 μL of DDH_2_O, and wash the bladder three times. Inject 100 μl of Poly-L-lysine Solution and let it stand for 20 min. Inject 100 μl of UM-UC-3 cells (1 × 10^6^) and let it stand for 2 h. After the completion of the modeling, bladder instillation was started on the 5th day, three times a week and used intravital imaging to observe tumor size. NCG mice (Strain NO. T001475) (5-week-old) were purchased from GemPharmatech company and bred under specific pathogen-free conditions. The bladder tumor specimens were divided into 5 mm * 5 mm tumor blocks and implanted under the skin of NCG mice. Passage them after they grow to 1500 mm^3^. The first two generations will not be used for experimental research. When the subcutaneous tumors of the third-generation NCG mice grew to 100 mm^3^, intraperitoneal injection of drugs was started, three times a week.

**Statistical analysis**

All data were presented as the mean ± standard deviation (SD) of the mean from at least three independent experiments. Statistical analyses were performed using GraphPad Prism 9 software. The significance of differences between groups was estimated using the Student’s t-test, chi-square test, or Wilcoxon test, as appropriate. Two-way Repeated Measures ANOVA was used to analyze the correlation between mouse tumor volume and body weight. Survival outcomes were assessed using Kaplan-Meier analysis with log-rank tests based on optimal cutoffs of TFRC and RRM1 expression in the GEO dataset. Combined prognostic effects were evaluated by stratifying patients into four expression subgroups. Simple linear regression models were used to evaluate the linear relationship between RRM1 or TFRC expression and gemcitabine resistance stage, and R² was calculated as a goodness-of-fit indicator. All statistical analyses were performed using two-tailed *p* values, and the statistical significance threshold was set at 0.05 if not explicitly mentioned.

**Colony formation assay**

Bladder cancer cells were seeded in 6-well plates for 24 h. Cells were then treated with Bava at different concentrations and incubated in a humidified incubator at 37 °C for 48 h. Continue incubation in the incubator for 12 days. Wash with PBS 3 times, fix with 4% paraformaldehyde for 1 h, then wash with PBS 3 times. Stained with crystal violet for 1 h, washed three times with PBS.

**Migration and invasion assay**

Cell migration assays were performed in a transwell chamber with an 8 μM pore (Corning, #353097). For invasion assay, we need to add the matrix gel to the transwell chamber at 37 °C for 0.5 h in advance. Cells were treated with or without Bava for 24 h. Cells were resuspended in serum-free medium and then added to transwell chamber, while the lower chamber contained fresh medium with 10% FBS for 24 h at 37 °C.

**Cell cycle assay**

Bladder cancer cells were treated with or without Bava for 24 h. Wash twice with pre-chilled PBS, fix with 70% cold ethanol at -20 °C for 24 h, and stain with 50 μg/mL PI and RNase A for 30 min. Use a flow cytometry instrument for cell cycle detection.

**FerroOrange assay and Total Iron Content Colorimetric Assay**

Bladder cancer cells were treated with or without Bava for 24 h.Wash the cells three times with HBSS, add FerroOrange or Mito-FerroGreen working solution (F374 or M489, dojindo) with a concentration of 1 µM or 5 µM, and incubate in a 37 °C, 5% CO2 incubator for 30 min. Use a fluorescent microplate reader or fluorescence microscope to observe. Bladder cancer cells were treated with or without Bava for 24 h. Wash twice with pre-chilled PBS, discard the PBS, add 200 μL of lysis solution, shake vigorously or vortex for 20-30 s, place on a shaking table for lysis for 2 h, and centrifuge the supernatant for subsequent determination of iron ion concentration. Mix the buffer and 4.5% potassium permanganate solution at a ratio of 1:1 and add to the sample, and incubate at 60 °C for 1 h. Add 30 μL of iron ion detection reagent, mix well, incubate at room temperature for 30 min, and centrifuge to take the supernatant. Absorbance was measured at 550 nm (E1042, Applygen Technologies Inc.).

**ATP content Assay**

Bladder cancer cells were treated with or without Bava for 24 h. Add 200 μL of lysis solution to the cell pellet, lyse and centrifuge, and take the supernatant. Add ATP detection working solution and leave it at room temperature for 3-5 min. Use a chemiluminescence instrument to measure the RLU value (S0026, Beyotime).

**Mitochondrial respiratory chain complex activity detection**

Mitochondrial respiratory chain complex I / NADH-CoQ reductase activity detection:Bladder cancer cells were treated with or without Bava for 24 h. Add 1 mL of extraction solution 1 to the cell pellet, and homogenize quickly 30 times on ice with a homogenizer. Centrifuge to get the supernatant, and centrifuge the supernatant again. The supernatant is the cytoplasmic extract. Add 200 μL of extraction solution 1 and 200 μL of extraction solution 2 to the precipitate, and crush it with ultrasonic waves. Add the detection working solution and detect the absorbance value at 340 nm. .Water bath at 37 ℃ for 1 min, and detect the absorbance value at 340 nm again (BC0515, Solarbio Life Science).

Mitochondrial respiratory chain complex II / succinate-CoQ reductase activity assay:Repeat the above steps, add the detection working solution, and detect the absorbance value at 605 nm. Water bath at 37 °C for 5 min, and detect the absorbance value at 605 nm again (BC3235, Solarbio Life Science).

Mitochondrial respiratory chain complex III / CoQ-cytochrome C reductase activity detection:Repeat the above steps, add the detection working solution, and detect the absorbance value at 550 nm. Water bath at 37 °C for 2 min, and detect the absorbance value at 550 nm again (BC3245, Solarbio Life Science).

Mitochondrial respiratory chain complex IV / cytochrome C oxidase activity detection:Repeat the above steps, add the detection working solution, incubate at 37 °C for 15 min. Detect the absorbance value at 550 nm, and detect again after 1 min (BC0945, Solarbio Life Science).

Mitochondrial respiratory chain complex V / ATP synthase activity test:Repeat the above steps, mix the sample with the detection working solution, and keep in a water bath at 37 °C for 30 min. Then add the working solution and mix well. Centrifuge at 8000 rpm for 10 min at room temperature. Take the supernatant and determine the phosphorus. Mix well and wait in a water bath at 40 °C for 10 min. Measure at 660 nm. Absorbance value (BC1445, Solarbio Life Science).

**Pull-down assay and LC-MS/MS**

Bava and CNBr-activated Sepharose 4B beads were incubated overnight at 4 °C, washed 5 times with wash buffer, and centrifuged. Add cell lysate and incubate overnight at 4 °C, wash 5 times, and centrifuge. Add 50 μL 2X loading buffer. Perform page gel separation and use Coomassie blue staining (17043001, Cytiva). The gel pieces containing proteins were cut, digested and analyzed by LC/MS-MS on an Orbitrap-Elite mass spectrometer (Thermo Scientific).

**Drug Affinity Responsive Target Stability (DARTS)**

Incubate the cell lysate with Bava (100 μM) at room temperature for 1 h. Add different concentrations of Pronase E and incubate at room temperature for 30 min. Add 5X loading buffer. Perform page gel separation, use Coomassie blue staining or continue to transfer to membrane for blocking, and use primary and secondary antibodies in the shell.

**Cellular Thermal Shift Assay (CETSA)**

Add DMSO or Bava (100 μM) to bladder cancer cells and treat for 6 h. Collect the cell pellets and divide them into 6 equal parts. Incubate them at different temperatures on a PCR instrument for 5 min each. Incubate the samples in liquid nitrogen and 37 °C. Repeat lysing 3 times. Centrifuge and take the supernatant and add 5X loading buffer. Use Page gel for separation and detection.

**Molecular docking and Molecular Dynamics**

Molecular docking technology was used to carry out to dock ligands into a protein’s binding site. The crystal structure of TFRC and EGFR (PDB code 1CX8 and 1M17) was derived from Protein DataBank repository (http://www.rcsb.org/pdb/). Bavachalcone was used as the ligand in this experiment, of which the ligand structure was generated by ChemDraw software (CambridgeSoft). Docking experiments were performed using the Schrodinger Maestro software. The 3D structure of TFRC-Bava or EGFR-Bava complex and hydrogen bond binding sites among them were visualized with Schrodinger.

The MD simulations were carried out using Desmond simulation package of Schrödinger 2021-2. Simulation systems were prepared by placing the structure of the TFRC and EGFR in a cubic simulation box with a buffer distance of 10Å in order to create a hydration model, and Na^+^ and Cl^−^ ions were added to neutralize the system and achieve physiological concentration (150 mM). The systems were parameterized with the OPLS_4 force field with TIP3P water. The NPT ensemble available within the Desmond package was used for minimization and relaxation of system. Each simulation was run for a total of 100 ns with a recording interval of 100 ps. The temperature and pressure were kept constant at 300 K and 1.01325 bar, respectively, throughout the simulations.

**Hematoxylin and Eosin (H&E) Staining, Immunofluorescence assay and Immunohistochemistry (IHC) staining**

H&E: Place the slices in xylene and soak them multiple times. Then soak them in absolute ethanol for 5 min, and then soak them in 95%, 85%, and 70% ethanol for 5 min each to achieve full hydration. Soak and wash 3 times with PBS solution. Add hematoxylin staining solution and stain thoroughly for 10 min. Wash with distilled water, differentiate with 1% hydrochloric acid ethanol, and then wash with double distilled water. Add blue-promoting liquid to reverse blue and wash with double-distilled water several times. Then add eosin staining solution for full staining for 3 min, and use different concentrations of ethanol for gradient dehydration. Soak in xylene twice and seal with neutral gum.

IF: Fix the sections with 4% paraformaldehyde for 1 h. Wash three times with PBS, permeabilize with PBS containing 0.3% Triton X-100, and block. Wash three times. Add the corresponding primary antibody, incubate at 4 °C overnight, add the corresponding fluorescent secondary antibody, and observe with a confocal fluorescence microscope.

IHC: Place the slides in an oven for 15 minutes, transfer to a xylene solution. Remove excess liquid and place in anhydrous ethanol. Place in 90% and 80% ethanol for 3 min. Rinse gently with dd H2O and soak in PBS for further hydration. Boil the slides in 10 mM sodium citrate buffer and cool the slides for 30 min. Wash the slides three times with ddH2O and incubate in a 3% hydrogen peroxide solution. Wash slides twice with ddH2O. Wash sections with wash buffer for 5 min. Block at room temperature for 1 h. Add corresponding primary antibody. Incubate at 4 °C overnight. Add corresponding secondary antibody, wash three times, and then perform DAB color development and counterstaining.

**Comet assay**

Bladder cancer cells were washed with pre-cooled PBS and centrifuged. Three layers of gel were prepared: the first layer used 1% normal melting point agarose; the second layer used 0.7% low melting point agarose LMA and cells; the third layer used 0.7% low melting point agarose LMA. After solidification, the gel was placed in pre-cooled Lysis Bufffer for 2 h, washed, and DNA electrophoresis was performed to unwind the DNA. Then 0.4 mM Tris-HCl (pH 7.5) buffer was added at 4 ℃ for neutralization. Cells were observed under a fluorescence microscope at a wavelength of 515~560 nm (KGA1302, Keygene Biotech).

**Co-IP analysis**

Cells transfected with indicated plasmids were harvested and then added with 500 μL Co-IP lysis buffer (20 mM Tris-HCl, 100 mM NaCl, 0.5 mM EDTA, 0.5% NP40 1 mM PMSF, 1x protease inhibitor) and cultivated on ice for 1 h. Thereafter, the mixture was centrifuged at 12000 g for 15 min at 4 °C. At the same time, 40 μL Anti-HA Affinity Beads or Anti-DYKDDDDK Affinity Beads (SA068005 or SA042005, Smart-Life sciences) were washed 5 times, the beads were added into cell lysis, which was rotated overnight at 4 °C. The beads were washed 5 times and boiled with loading buffer. Finally the bounded proteins were determined by SDS-PAGE.

**RNAseq**

Total RNA was extracted from DMSO or Bava treated cells and was submitted to the Shanghai OE Biotech Co., Ltd for RNA sequencing. The genes with log fold change ≥ 1 and p value < 0.05 was defined as differentially expressed genes (DEGs). Volcano plot and heatmaps were clustered by the bioinformatics online (http://www.bioinformatics.com.cn/). The gene ontology (GO) enrichment (biological process (BP), molecular function (MF), and cell component (CC)) analysis was performed by the Gene Ontology Resource (http://geneontology.org/). The enrichment analysis of the KEGG pathway was conducted by the KOBAS 3.0. (http://kobas.cbi.pku.edu.cn/kobas3). Gene set enrichment analysis (GSEA) was performed using the GSEA software 4.1.

**Membrane and cytosol protein extraction**

Bladder cancer cells were treated with or without Bava or Gem for 24 h. Add EGF to activate for 30 minutes. Collect the cell pellet and wash it three times with pre-cooled PBS. Add membrane protein extraction reagent and lyse on ice. Centrifuge to obtain the supernatant and centrifuge again. The supernatant is the cytoplasmic protein. Add membrane protein extraction reagent to the pellet, vortex, lyse on ice, and centrifuge again to obtain the supernatant, which is the membrane protein.

**Western blot assay**

Cells and tissues protein were lysised and extracted by RIPA buffer (R0030, solarbio life science). Total protein levels were determined by BCA assay (cat. no. 23227, Thermo Fisher; CA, USA). Proteins (15 μg) were separated by 10% or 8% SDS-PAGE and electrotransferred onto polyvinylidene fluoride membranes. Membranes were blocked in 5% non-fat milk for 1.5 h. Membranes were then probed with a suitable primary antibody. Immunoreactive bands were detected by incubating with specific secondary antibodies conjugated to horse-radish peroxidase and enhanced chemiluminescence reagent.

**Quantitative reverse transcription-PCR (qRT-PCR)**

Total RNA was isolated from bladder cancer cells using the EZ-press RNA Purification Kit (B0004D, EZBioscience). The EZ-press Cell to cDNA Kit PLUS II (B0003C, EZBioscience) was used for reverse transcription. qRT-PCR was conducted using the 2× EZ Color SYBR Green qRT-PCR Master Mix (CQ22, EZBioscience).

**Luciferase reporter assay**

DNA transfection and luciferase assays were carried out following the Dual luciferase reporter assay system (E1910, Promega). HEK293T cells were cultured in 24-well plate one day before transfection with 1 × 10^5^ cells per well. The Renilla plasmid (pRL-TK) was transfected with indicated vectors and firefly luciferase activity obtained from each sample was normalized to the Renilla luciferase activity from the same sample.

**CHIP**

Add 37% formaldehyde to the cells for cross-linking, at room temperature for 10 min, add 10X glycine to quench the unreacted formaldehyde. Wash with pre-cooled PBS three times, scrape the cells and centrifuge, add lysis buffer, and ultrasonically break the DNA to the appropriate fragment length on ice. Centrifuge again to take the supernatant and combine it with protein G agarose at 4 ℃ for 2 h. Collect the supernatant by centrifugation, add the antibody and combine it at 4 ℃ overnight, then add protein G agarose beads. Centrifuge and take the supernatant for multiple elutions. Add NaCl at 65 ℃ overnight, then add ribonuclease A and incubate at 37 ℃ for 30 min. Then add 0.5 M EDTA, 1 M Tris-HCl and proteinase K, and react at 45 ℃ for 2 h. Finally, use a centrifugal column to purify the DNA. Perform PCR or qRT-PCR analysis.

**Supplementary figures:**


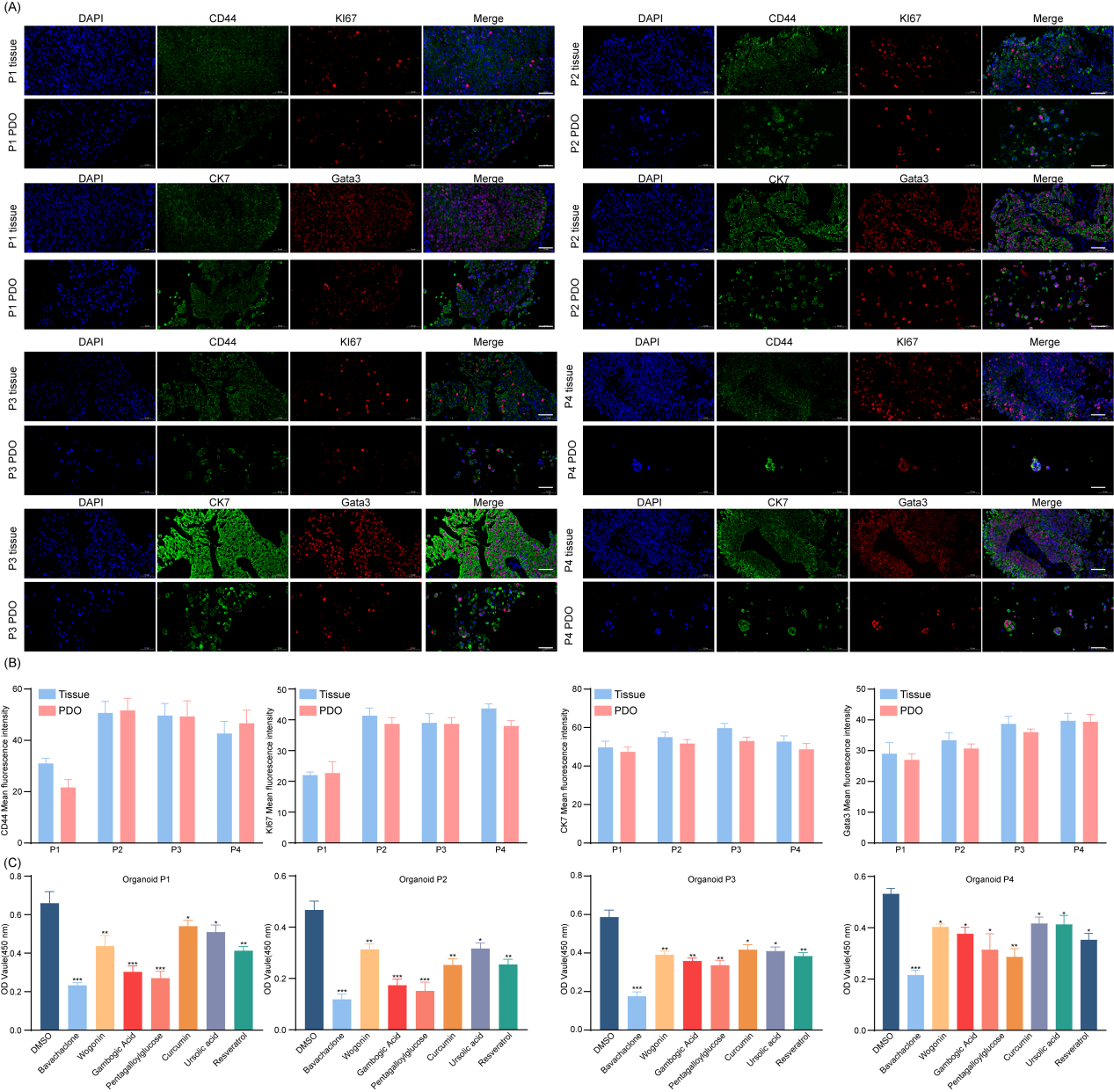


**Figure S1. Bladder cancer organoid mIHC staining.** (A) mIHC staining of four bladder cancer organoids. Scale bars, 50 μm. (B) Quantification of fluorescence intensity in tumor tissue and patient-derived organoids. (C) Quantification of organoid viability by CCK-8 assay following drug treatment. Data represent the mean ± SD of three replicates. **p* < 0.05, ***p* < 0.01 and ****p* < 0.001.


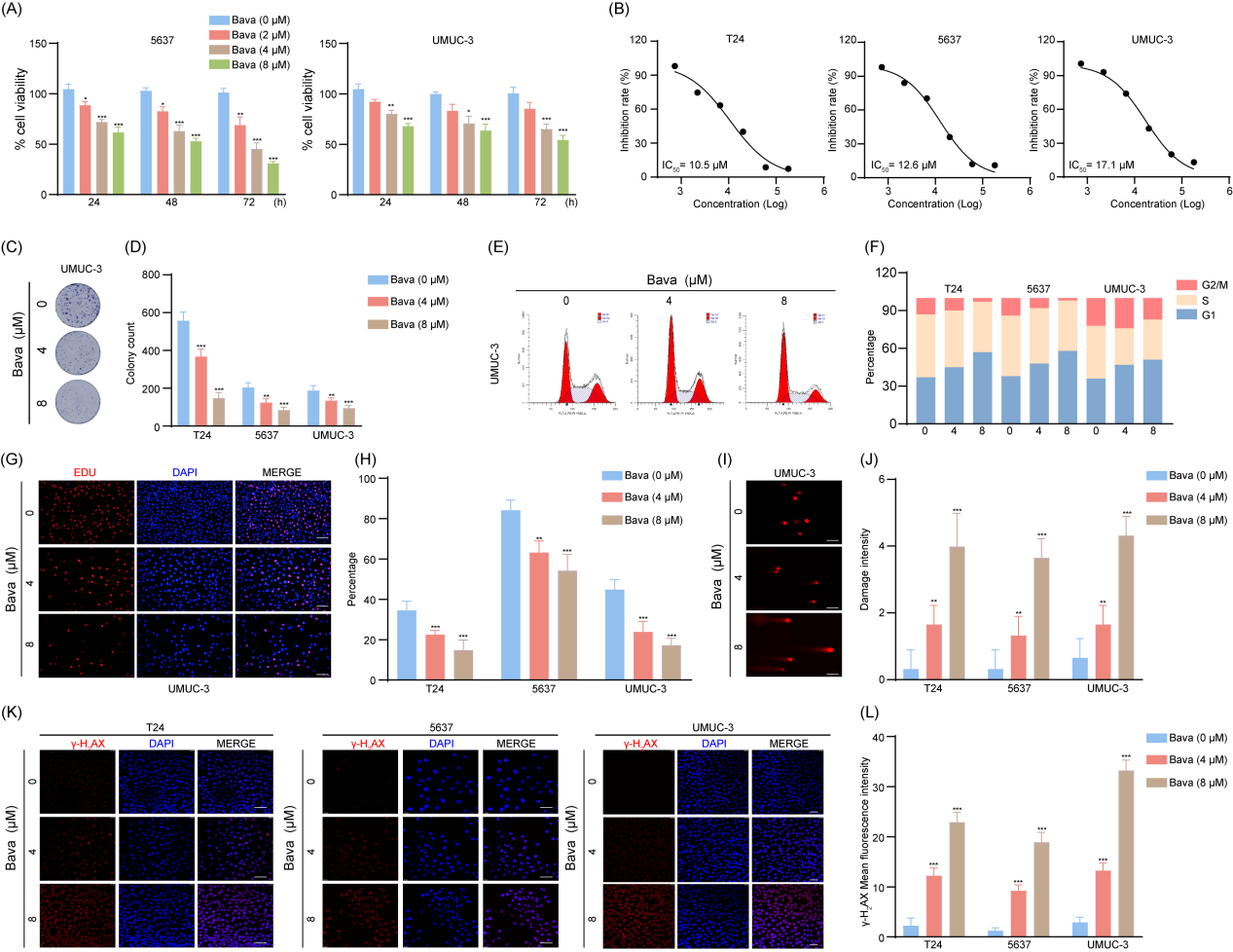


**Figure S2.** **In vitro study on the inhibition of bladder cancer by Bavachalcone.** (A) The inhibitory effect of Bava on the proliferation of 5637 and UMUC-3 cells at various concentrations and time points. (B) IC50 values of Bava in T24, 5637, and UMUC-3 cells at 24 h. (C) Representative clonogenic assay images of UMUC-3 cells after 24 h incubation with Bava. (D) Quantification of colony formation in T24, 5637, and UMUC-3 cells after 24 h incubation with Bava. (E) Representative flow cytometry histograms showing the cell cycle distribution of UMUC-3 cells after 24 h treatment with Bava. (F) Quantification of cell cycle distribution in T24, 5637, and UMUC-3 cells after 24 h treatment with Bava. (G) Representative immunofluorescence images showing DNA replication in UMUC-3 cells following Bava treatment. Scale bar, 100 μm. (H) Quantification of DNA replication in T24, 5637, and UMUC-3 cells following Bava treatment. (I) Representative immunofluorescence images showing DNA damage in UMUC-3 cells after 24 h incubation with Bava. Scale bar, 100 μm. (J) Quantification of DNA damage in T24, 5637, and UMUC-3 cells after 24 h incubation with Bava. Scale bar, 100 μm. (K) Representative immunofluorescence images showing γ-H2AX expression in UMUC-3 cells following Bava treatment. Scale bar, 50 μm. (L) Quantification of γ-H2AX expression in T24, 5637, and UMUC-3 cells following Bava treatment. Data represent the mean ± SD of three replicates. **p* < 0.05, ***p* < 0.01 and ****p* < 0.001.


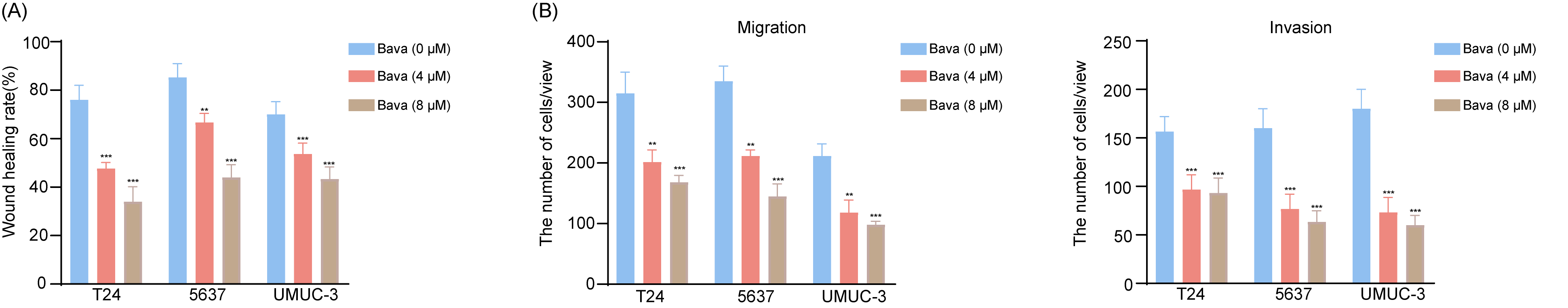


**Figure S3.** **In vitro study on the inhibition of bladder cancer by Bavachalcone.** (A) Quantification of wound closure in T24, 5637, and UMUC-3 cells after 24 h treatment with Bava using a wound-healing assay. (B) Quantification of invasion and migration of T24, 5637, and UMUC-3 cells after 24 h treatment with Bava using transwell assays. Data represent the mean ± SD of three replicates. ***p* < 0.01 and ****p* < 0.001.

**
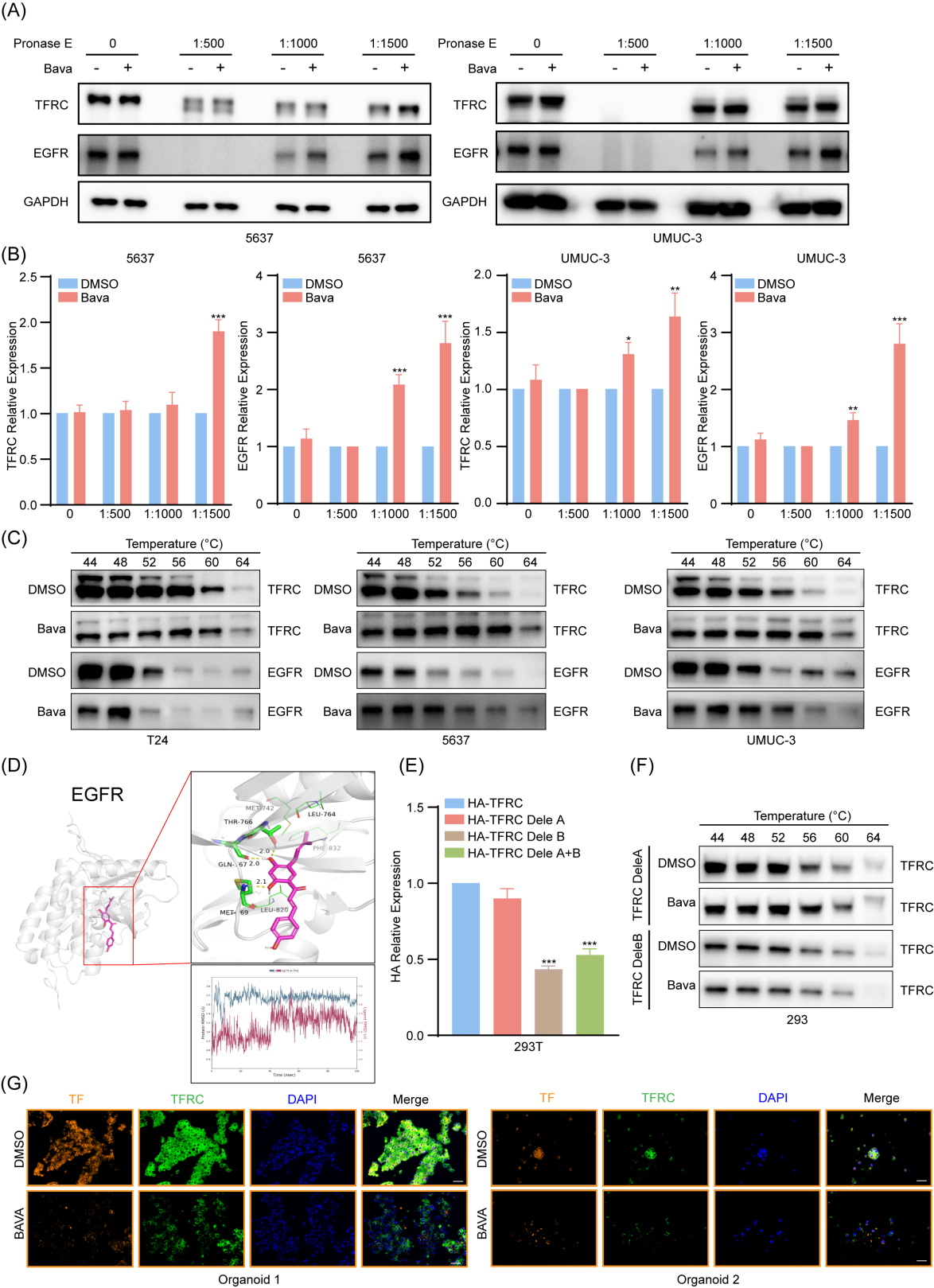
**

**Figure S4.** **Bavachalcone directly targets TFRC and EGFR.** (A-B) Representative immunoblot images showing that Bavachalcone reduces pronase E–mediated hydrolysis of TFRC and EGFR in DARTS assays, with quantification of band intensities. (C) Representative immunoblot showing that Bavachalcone stabilizes TFRC and EGFR in CETSA assays, preventing their thermal denaturation. (D) Orthogonal view of the Bavachalcone–EGFR complex in the binding pocket, with accompanying molecular dynamics simulation. (E) Quantification of immunoblot band intensities from figure 3E. (F) Representative immunoblot showing that Bavachalcone fails to stabilize TFRC in CETSA assays after deletion of residues 416–460. (G) Representative immunofluorescence images showing co-localization of transferrin and TFRC in bladder cancer organoids before and after Bavachalcone treatment. Scale bar, 50 μm. Data represent the mean ± SD of three replicates. **p* < 0.05, ***p* < 0.01 and ****p* < 0.001.


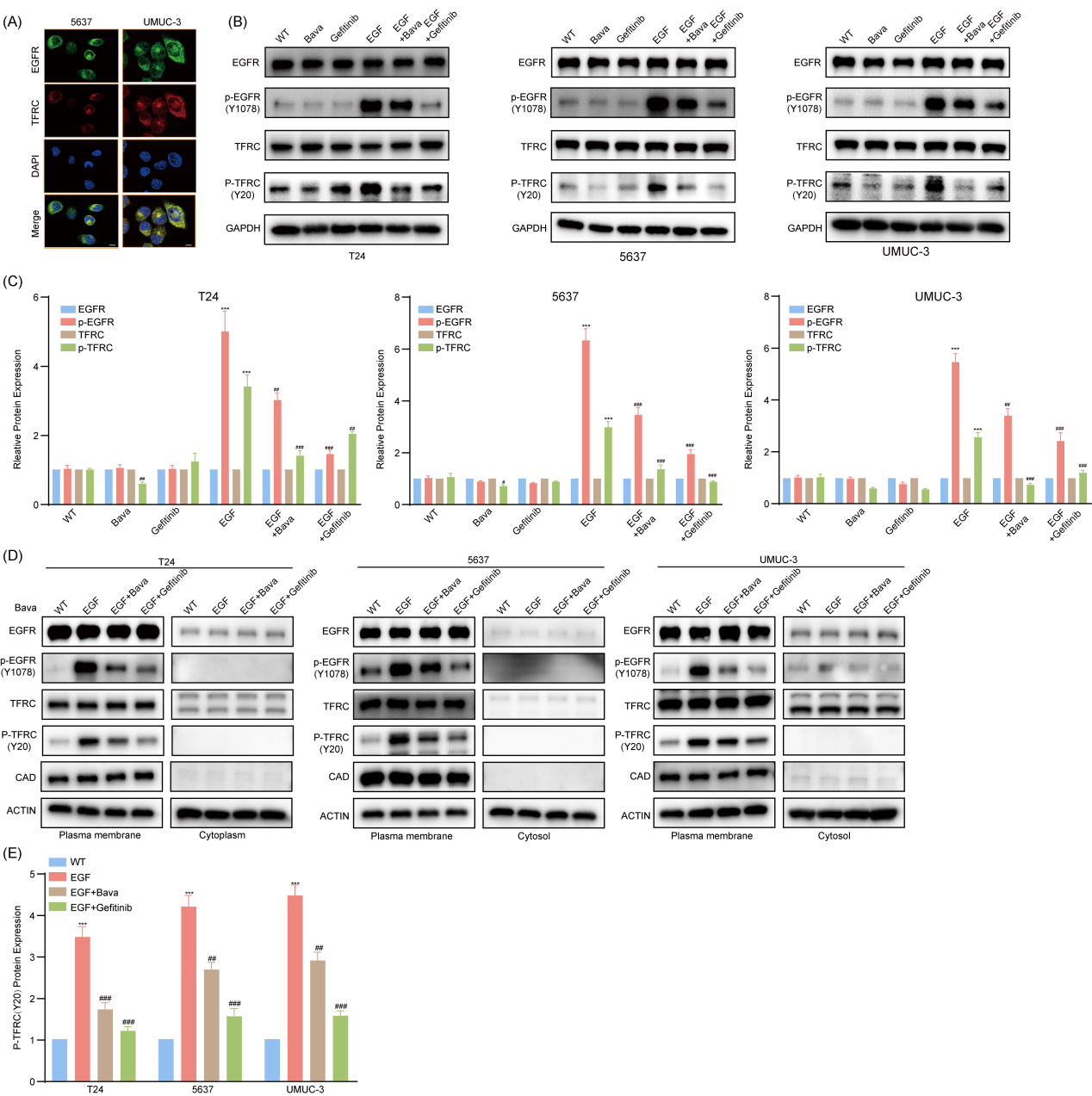


**Figure S5.** **Bavachalcone inhibits EGF-induced EGFR and TFRC phosphorylation.** (A) Representative immunofluorescence images showing localization of TFRC and EGFR in 5637 and UMUC-3 cells. Scale bar, 25 μm. (B-C) Representative immunoblot images (B) and quantification (C) of EGFR and TFRC phosphorylation in the absence or presence of EGF stimulation, and the inhibitory effects of Bavachalcone and gefitinib on their phosphorylation.

(D-E) Representative immunoblot images and quantification showing phosphorylation of EGFR and TFRC in membrane fractions of T24, 5637, and UMUC-3 cells. Data represent the mean ± SD of three replicates.****p* < 0.001. #*p* < 0.05, ##*p* < 0.01 and ###*p* < 0.001.


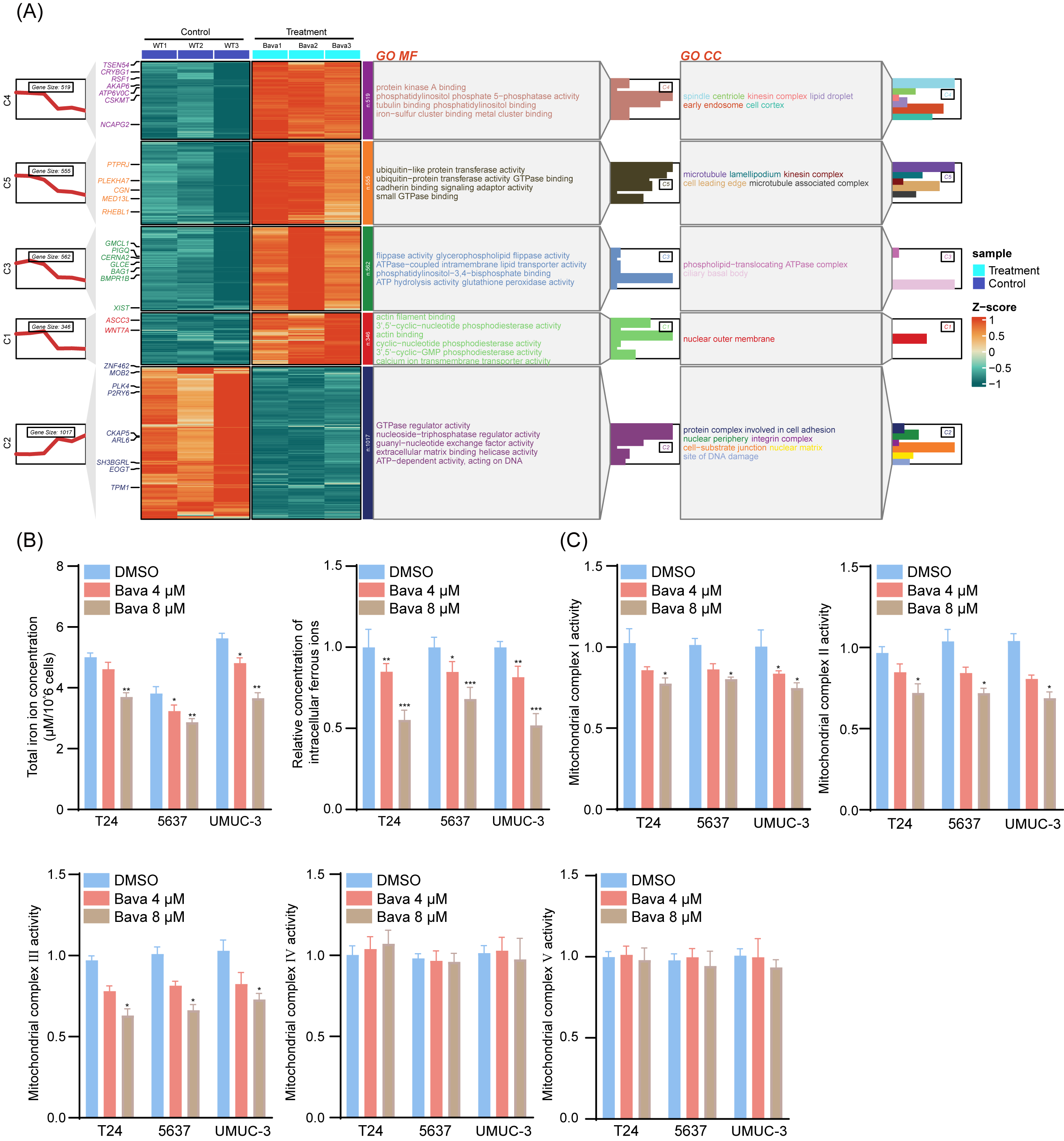


**Figure S6.** **Bavachalcone inhibits iron influx and mitochondrial respiratory chain activity in bladder cancer.** (A) Heatmap and GO enrichment analysis of representative genes and pathways altered in T24 cells before and after Bavachalcone treatment. (B) Quantification of total iron and Fe²⁺ levels in T24, 5637, and UMUC-3 cells treated with various concentrations of Bavachalcone. (C) Assessment of mitochondrial respiratory chain complex activities in T24, 5637, and UMUC-3 cells following treatment with different concentrations of Bavachalcone. Data represent the mean ± SD of three replicates. **p* < 0.05, ***p* < 0.01 and ****p* < 0.001.


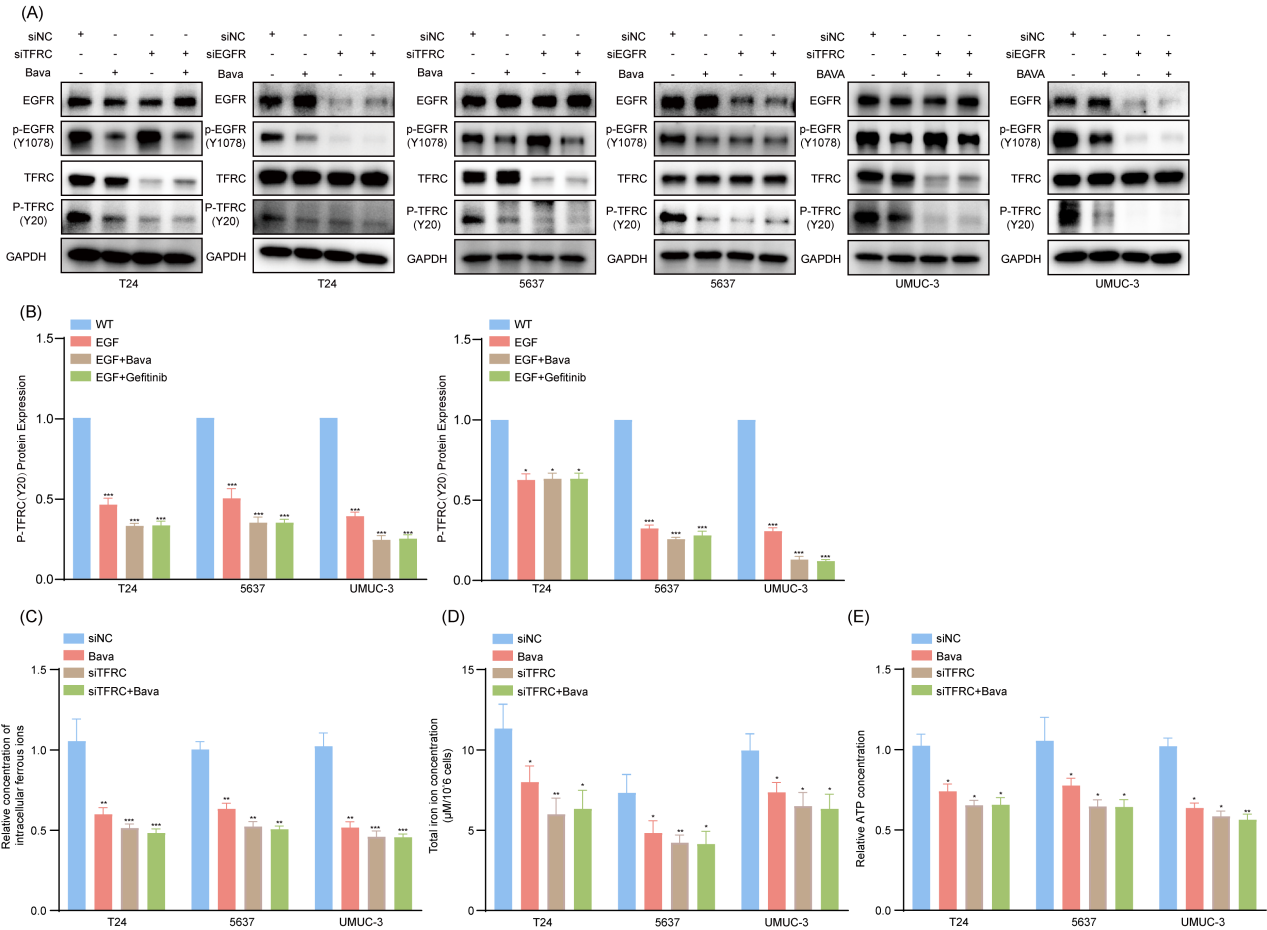


**Figure S7.** **Bava could not further inhibit the phosphorylation of EGFR and TFRC after knockdown of EGFR and TFRC.** (A-B) Representative immunoblot images and quantification showing EGFR and TFRC phosphorylation after TFRC or EGFR knockdown, with or without Bavachalcone treatment. (C-E) Intracellular total iron, Fe²⁺ levels, and ATP production were measured in TFRC-knockdown cells treated with or without Bavachalcone.

Data represent the mean ± SD of three replicates. **p* < 0.05, ***p* < 0.01 and ****p* < 0.001.


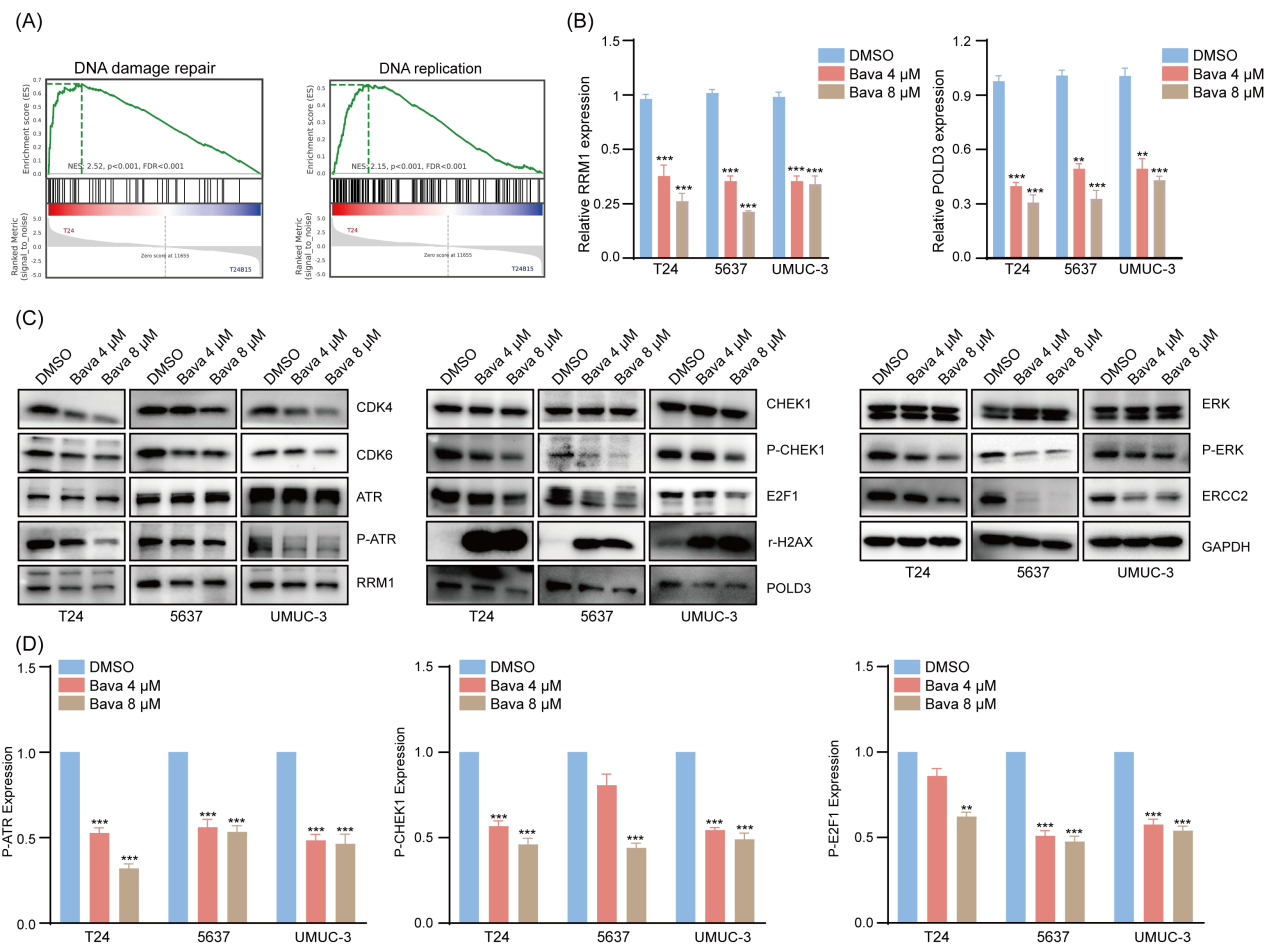


**Figure S8.** **Bavachalcone inhibits DNA damage repair in bladder cancer.** (A) GSEA of DNA damage repair and DNA replication pathways in T24 cells following Bavachalcone treatment. (B) Quantification of RRM1 and POLD3 mRNA levels in T24 cells after Bavachalcone treatment. (C-D) Representative immunoblot images and quantification showing changes in cell cycle, DNA damage repair, and DNA replication–related proteins in T24, 5637, and UMUC-3 cells following Bavachalcone treatment. Data represent the mean ± SD of three replicates. ***p* < 0.01 and ****p* < 0.001.

**
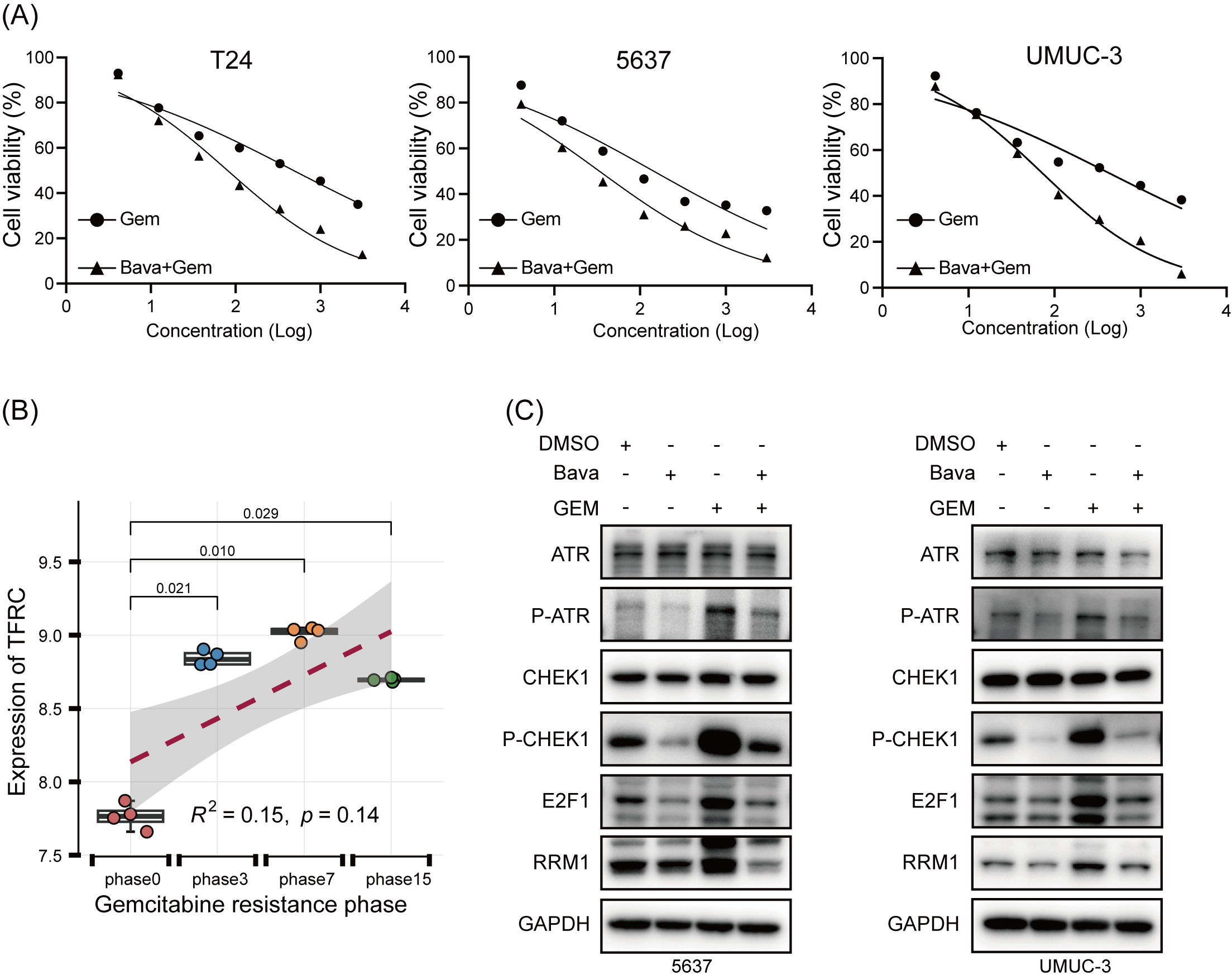
**

**Figure S9.** **Bavachalcone combined with gemcitabine inhibits DNA damage repair in bladder cancer.** (A) IC50 values of gemcitabine alone and gemcitabine plus Bavachalcone in T24, 5637, and UMUC-3 cells. (B) Analysis of TFRC expression during the progression of gemcitabine resistance using dataset GSE190636. (C) Representative immunoblot showing ATR-CHEK1-E2F1 pathway protein levels after treatment with Bavachalcone, gemcitabine, or their combination.


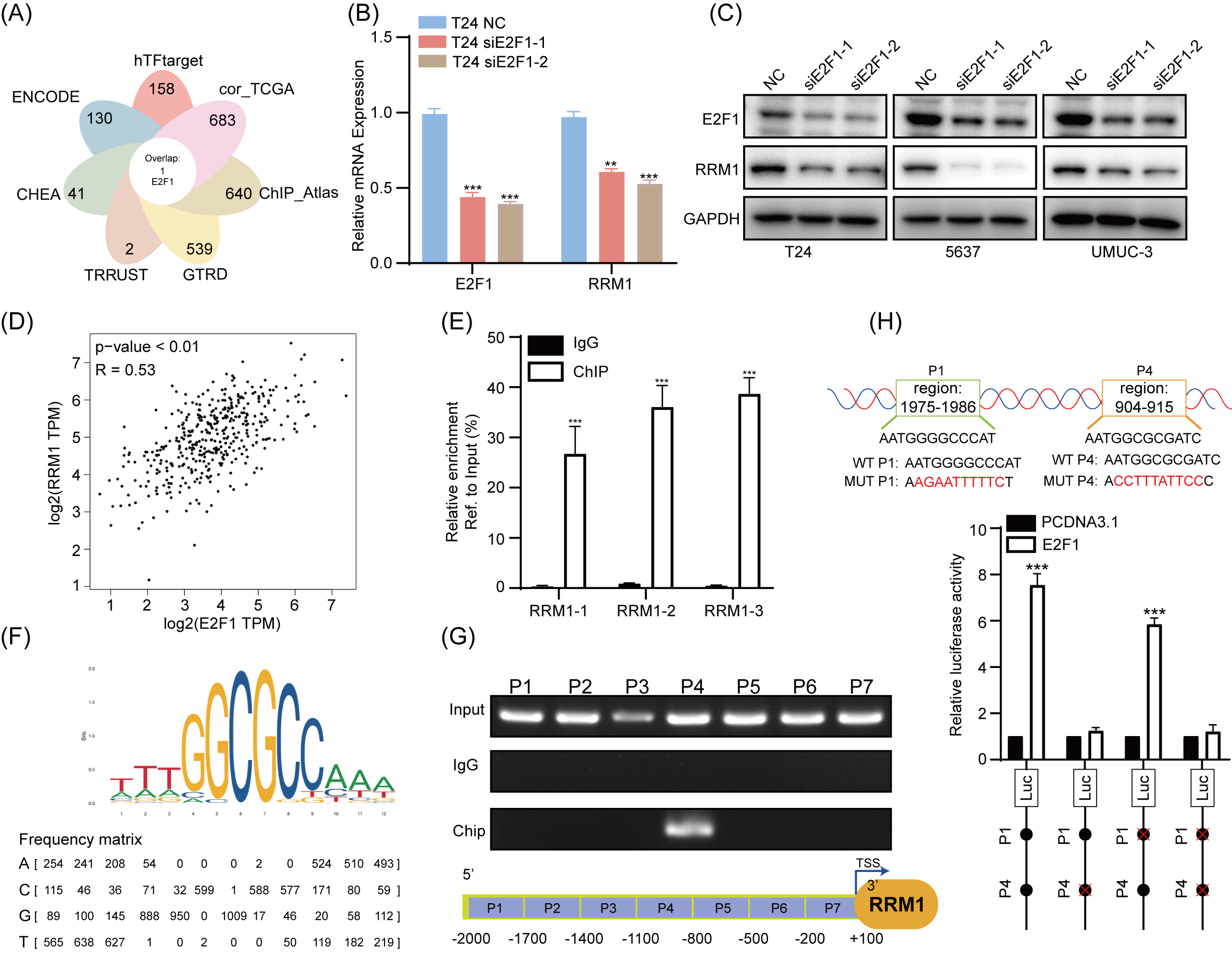


**Figure S10.** **E2F1 regulates the transcription of RRM1.** (A-B) Representative immunoblot images (A) and quantification (B) showing ATR-CHEK1-E2F1 pathway protein levels in cells pretreated with ferrous sulfate, followed by Bavachalcone or deferoxamine treatment. Data represent the mean ± SD of three replicates. **p* < 0.05, ***p* < 0.01 and ****p* < 0.001. #*p* < 0.05, ##*p* < 0.01 and ###*p* < 0.001.


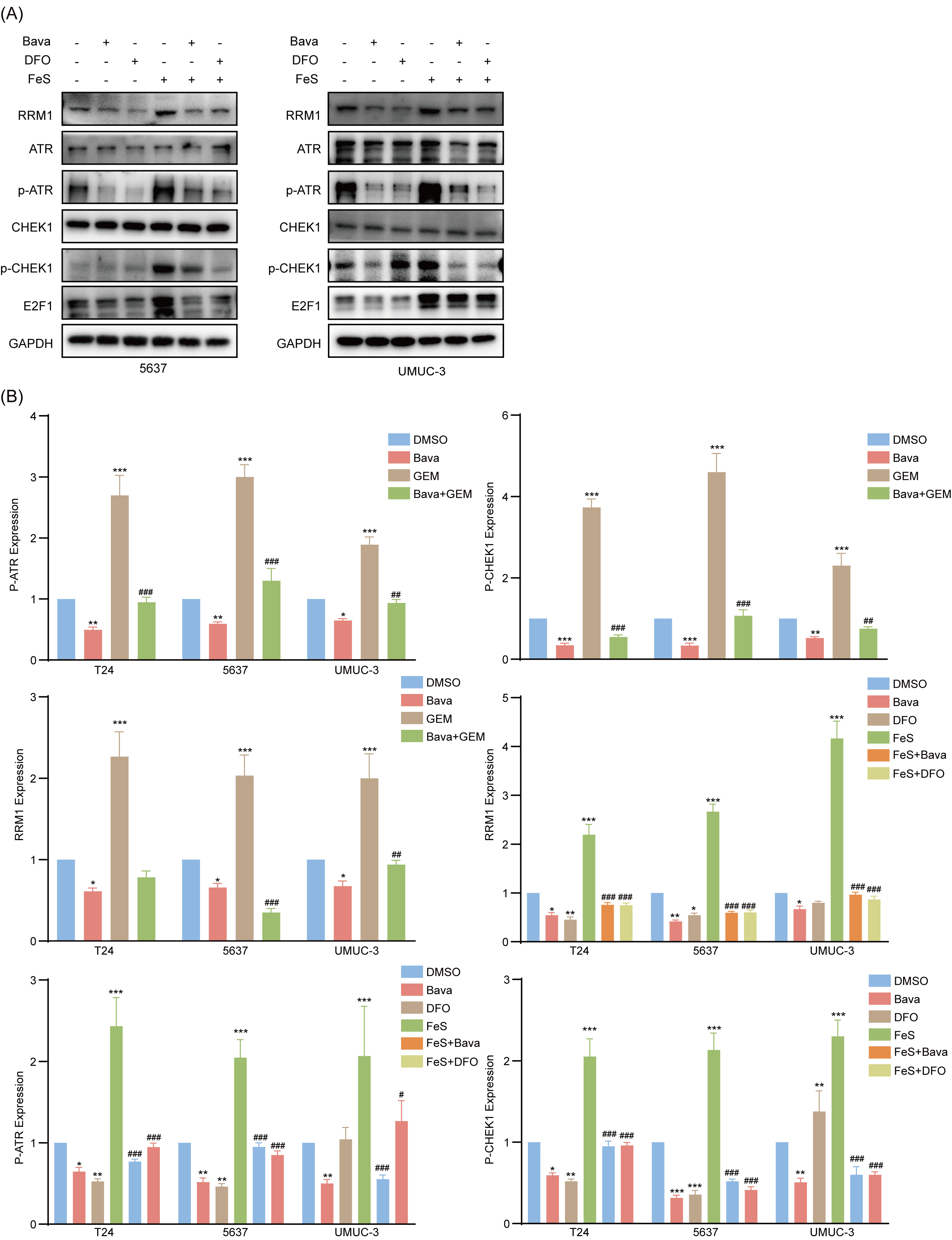


**Figure S11.** **Bavachalcone combined with DFO or FeS affects the iron-dependent ATR-CHEK1-E2F1 signaling pathway.** (A) Bioinformatics analysis identified E2F1 as a potential transcription factor regulating RRM1. (B-C) Quantification of RRM1 and E2F1 mRNA (B) and protein (C) levels following E2F1 knockdown. (D) Pearson correlation analysis showing a positive correlation between E2F1 and RRM1 mRNA expression in the TCGA bladder cancer dataset. (E) Chromatin immunoprecipitation followed by qPCR demonstrating E2F1 binding to the RRM1 promoter region. (F) Schematic of putative E2F1-binding sites within the RRM1 promoter. (G) ChIP-PCR validation of E2F1 binding to segmented fragments of the RRM1 promoter. (H) Dual-luciferase reporter assays showing that mutation of the E2F1-binding sites abolishes E2F1-mediated activation of the RRM1 promoter. Data represent the mean ± SD of three replicates. ***p* < 0.01 and ****p* < 0.001.

**
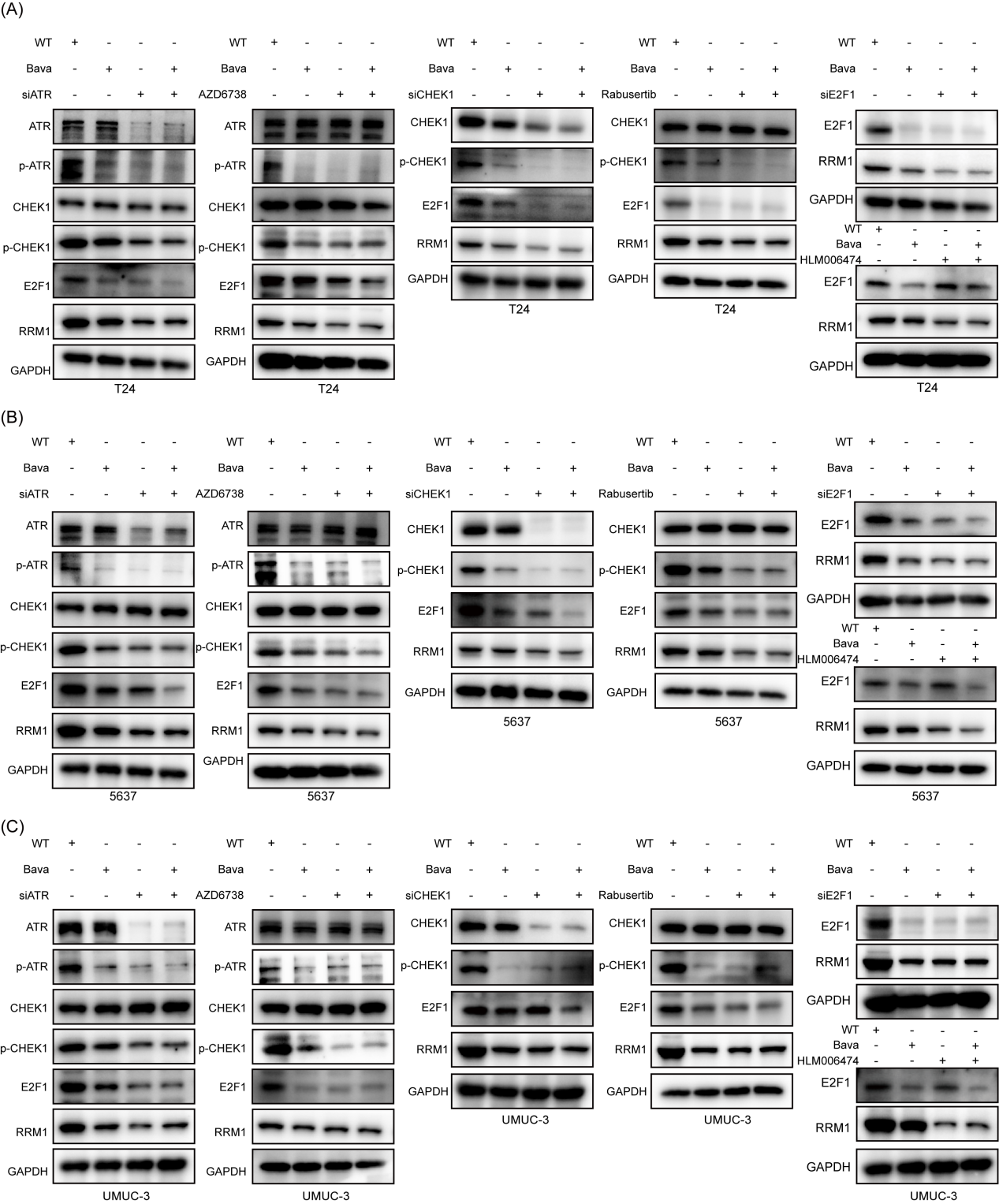
**

**Figure S12**. **Knockdown or pharmacological inhibition of ATR–CHEK1–E2F1 signaling modulates Bavachalcone’s pathway suppression.** (A-C) Representative immunoblot showing ATR, CHEK1, and E2F1 protein levels in T24 cells with or without Bavachalcone (Bava) treatment following siRNA-mediated knockdown of ATR, CHEK1, or E2F1. Representative immunoblot showing ATR, CHEK1, and E2F1 protein levels in T24 cells treated with Bavachalcone in combination with the ATR inhibitor AZD6738, the CHEK1 inhibitor Rabusertib, or the E2F1 inhibitor HLM006474.


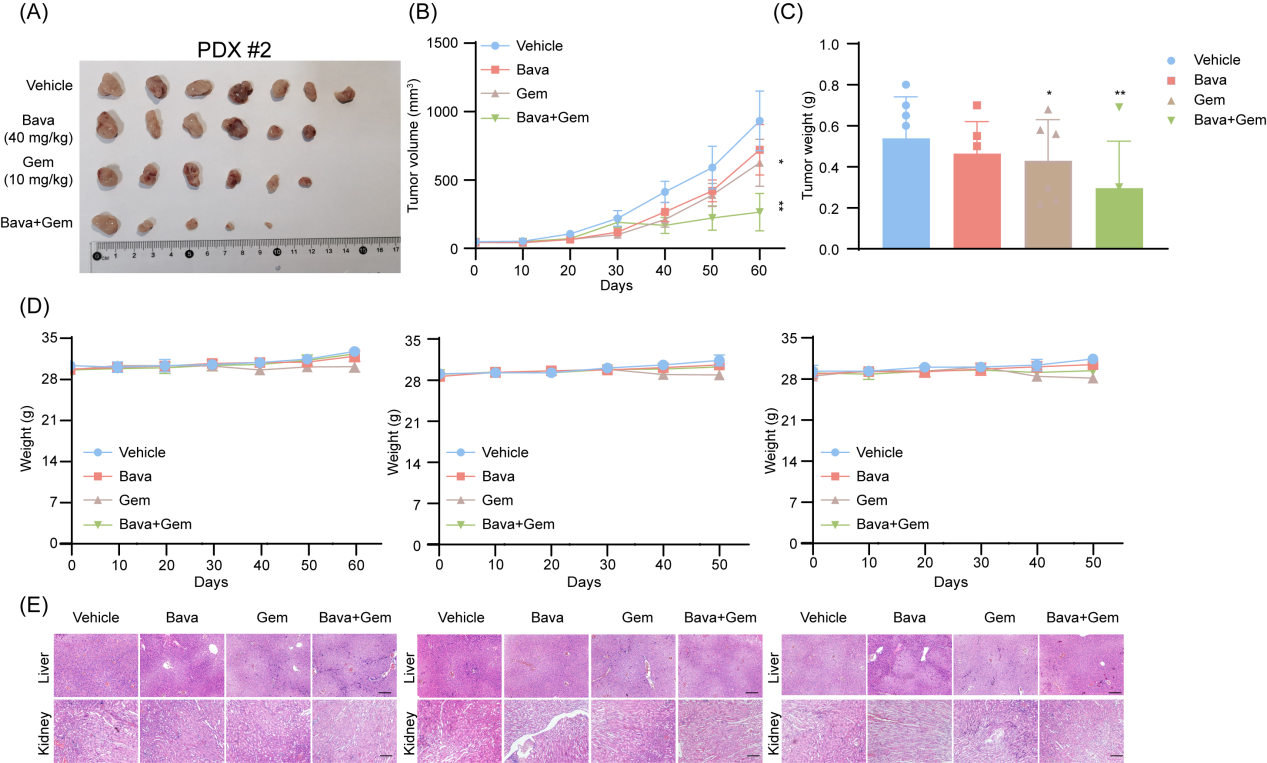


**Figure S13.** **Bavachalcone inhibits progression in bladder cancer PDX models.** (A-B) Representative images of tumors excised from subcutaneous tissue of PDX model mice after sacrifice. (C) Tumor weights of PDX models following excision. (D) Body weight changes of PDX mice over the course of the experiment. (E) Representative H&E staining of liver and kidney sections from PDX models. Scale bar, 100 μm. Data represent the mean ± SD of three replicates. **p* < 0.05 and ***p* < 0.01.


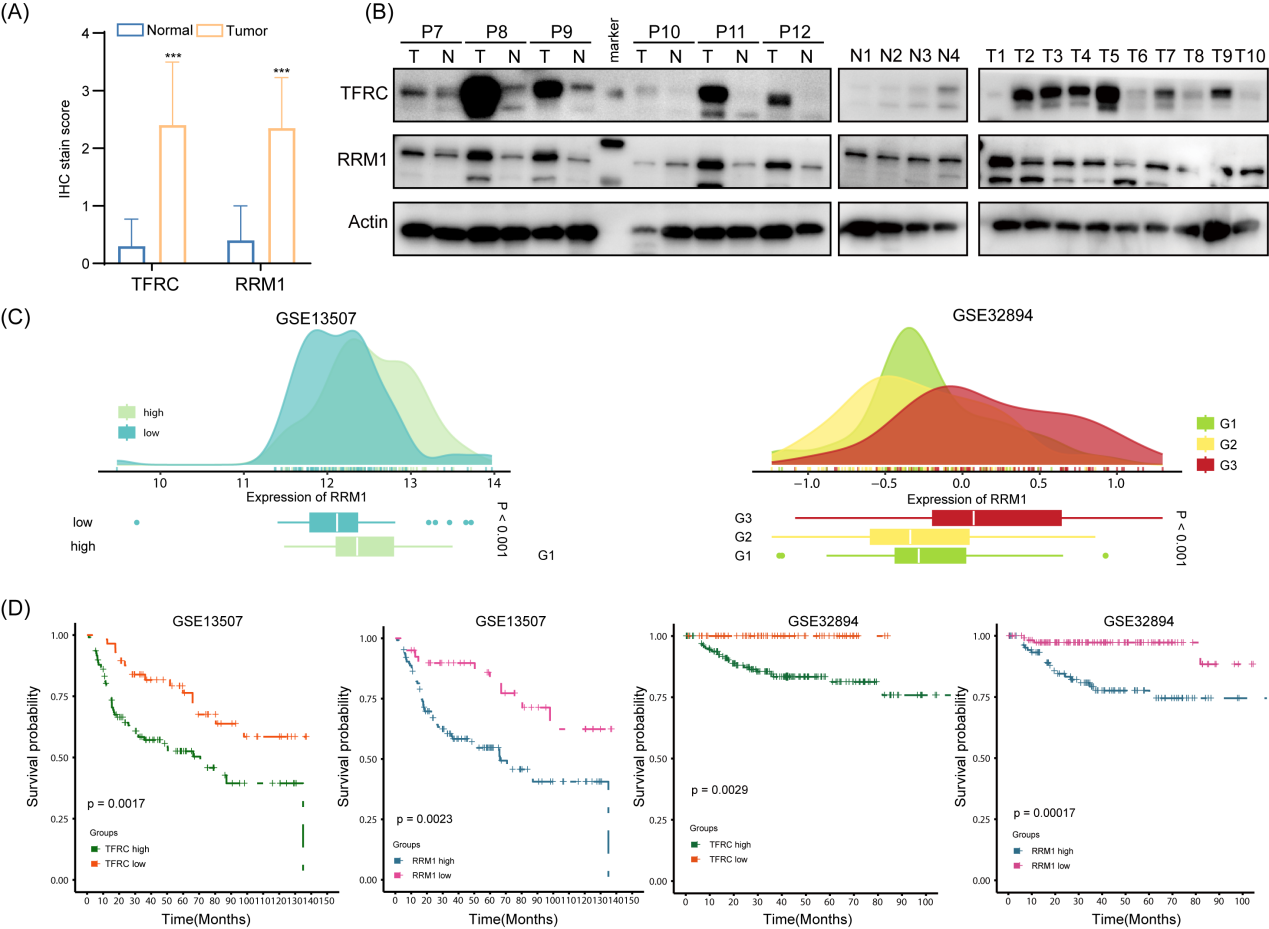


**Figure S14.** **TFRC and RRM1 are poor prognostic indicators in bladder cancer.** (A) Representative immunohistochemistry scores showing TFRC and RRM1 expression in normal bladder tissue and bladder cancer specimens across different stages. (B) Representative immunoblot showing TFRC and RRM1 protein levels in paired bladder cancer and adjacent normal tissues. (C) RRM1 expression levels at different tumor stages in the GSE13507 and GSE32894 datasets. (D) Association between TFRC and RRM1 expression and patient prognosis in the GSE13507 and GSE32894 datasets. Data represent the mean ± SD of three replicates. ****p* < 0.001.
